# Revisiting Theron’s hypothesis on the origin of fairy circles after four decades: Euphorbias are not the cause

**DOI:** 10.1186/s12862-021-01834-5

**Published:** 2021-05-28

**Authors:** Stephan Getzin, Ailly Nambwandja, Sönke Holch, Kerstin Wiegand

**Affiliations:** 1 Department of Ecosystem Modelling, University of Goettingen, Goettingen, Germany; 2 Department of Ecological Modelling, Helmholtz Centre for Environmental Research–UFZ, Leipzig, Germany; 3 Gobabeb-Namib Research Institute, Namib Naukluft Park, Gobabeb, Namibia

**Keywords:** Clark–Evans index, Clustered dispersal, Drone, Euphorbias, Fairy circles, Heterogeneity, Homogeneity, Infiltration, Pattern-process inference, Spatially periodic patterns

## Abstract

**Background:**

The Euphorbia hypothesis on the origin of fairy circles (FCs) in Namibia dates back to 1979. It proposes that the remains of decaying shrubs would induce an allelopathic interaction with the grasses and thereby cause bare-soil FCs. Here, we investigated this hypothesis based on revisiting marked Euphorbias after four decades, comparing the typical size distribution of dead *Euphorbia damarana* and FCs, and analyzing the spatial patterns of Euphorbias and FCs within the same drone-mapped study plots in three regions of Namibia.

**Results:**

We found four dead Euphorbias in the southern Giribes that were marked by G.K. Theron about 40 years ago. Those locations did not develop into FCs over this time span. However, for the four dead Euphorbias, we provide photographic evidence that grass tufts were growing at the metal pins of those decaying shrubs, agreeing with previous research findings that the soil taken from beneath dead *E. damarana* shrubs was stimulating rather than inhibiting the growth of grasses. In the Giribes, there were very large FCs that ranged in diameter from 13.0 to 19.1 m. By contrast, the measured dead *E. damarana*, including the largest plants that we could find, ranged in size only between 4.2 and 11.7 m. At Brandberg, we found particularly small FCs with diameters between 2.4 and 2.7 m but the dead *E. damarana*, including the smallest dead shrubs in the area, ranged in size between 4.1 and 7.2 m. Hence given these size mismatches, the decaying Euphorbias cannot induce such observed FCs in the two regions. Spatial patterns of *E. damarana* and FCs in the two regions Giribes and Brandberg, as well as of *E. gummifera* and FCs near Garub, showed a strong mismatch within the same habitat: in four out of five plots the patterns differed significantly. FCs were regularly distributed while Euphorbias were predominantly clustered.

**Conclusions:**

We reject the Euphorbia hypothesis based on the fact that grass growth was not prevented under decaying shrubs, the size of dead Euphorbias cannot explain the size of observed FCs and the spatial distribution of Euphorbias cannot cause the specific pattern signature of FCs.

**Supplementary Information:**

The online version contains supplementary material available at 10.1186/s12862-021-01834-5.

## Background

Fairy circles (FCs) are round grassland gaps that can be found along the Namib Desert in south-western Africa. At this transition from arid grassland to desert, FCs are found on nutrient-deficient sandy soils where just one or two grass species of the genus *Stipagrostis* predominate. These ecosystems are so species-poor because FCs are primarily confined to rainfall isohyets ranging between 50 and 100 mm mean annual precipitation [1]. In these arid grasslands along the Namib, FCs are characterized by having the unique ability to form spatially periodic patterns where each circle has not only on average six nearest neighbors, but importantly the distances to the six neighbors are approximately equal [2].

In Namibia, the typical mean diameters of the FCs increase with aridity from 3 to 4 m in the south to 6 or 10 m further to the northwest [1]. Some FCs can also be much larger with diameters exceeding 20 or 30 m, hence they are called “mega circles” [3]. FCs function as an extra source of water for the matrix vegetation because rain water in the bare gaps is not transpired by grasses and thus not reverted back to the atmosphere. In Namibia soil moisture inside the FCs can be, for example, five times higher than outside the FC, i.e. in the matrix [4]. The higher soil moisture is not only restricted to deep aeolian sands but can also be found in the gravel plains of the Central Namib [5].

**Fig. 1:**
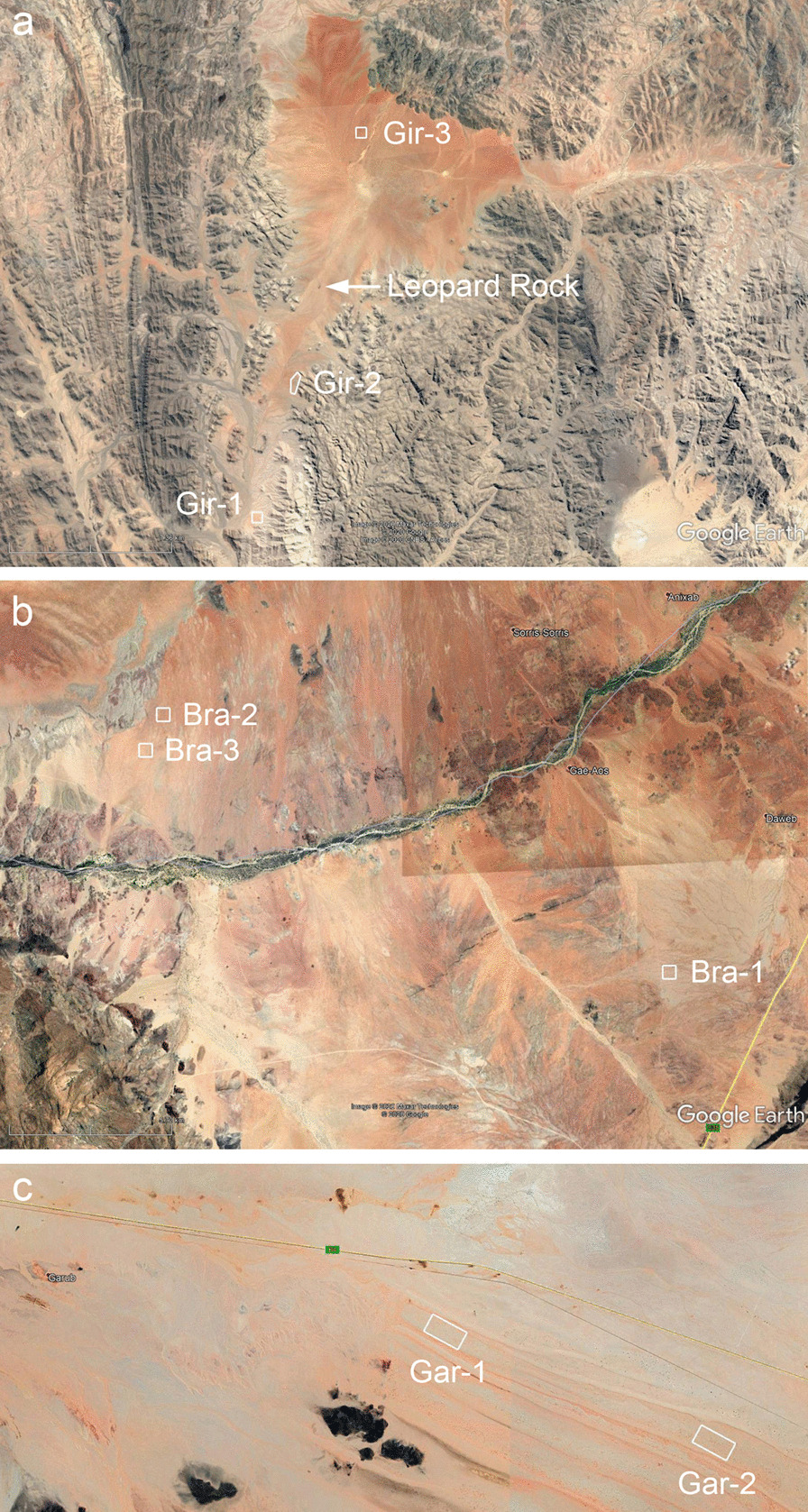
The three study regions of Namibia. Three plots were studied at the Giribes (**a**). North of the Leopard Rock, fairy circles dominate the landscape without any Euphorbia shrubs nearby. South of the rock, Euphorbias and FCs only rarely coexist in very isolated places along the mountain edges, as indicated by the plots Gir-1 and Gir-2. Three study plots were located around Brandberg, which is the highest mountain of Namibia (**b**). The ephemeral Ugab River runs north of Brandberg. In southern Namibia, two study plots were situated in the interdune valley near Garub (**c**). For location of these areas within Namibia, see Additional file 1: Fig. S1

Fairy circles in Namibia have been a mystery for decades. One of the earliest hypotheses about their origin was proposed in 1979 by G.K. Theron [6], who suggested that FCs in northern Namibia such as the Giribes Plains or the Marienfluss Valley result from an allelopathic effect from the remains of dead *Euphorbia damarana* shrubs on the grasses. He proposed that the decaying branches of the shrubs could leave some toxic matter behind in the sandy soil which prevents the growth of the grasses. While Theron marked several dead Euphorbias in the southern Giribes (for map see Fig. 1, Additional file 1: Fig. S1) with metal pins to establish a long-term monitoring experiment, he never published an own follow-up study on this specific experiment.

So far, many more hypotheses on the origin of fairy circles have been proposed. From the 1980s to the beginning of the new millennium, very little data existed and nearly nothing was known about the origin and maintenance of fairy circles. Cox [7] found a correlation between rodents like gerbils and FCs in the Central Namib and suggested that rodent activity would cause the bare patches. Moll [8] and later Becker & Getzin [9] found harvester termites around FCs. Picker et al. [4] and Juergens [10] found a correlation between FCs and ants and FCs and sand termites, respectively, and both suggested causality due to insect herbivory. Also, abiotic gas, possibly inducing plant chlorosis, has been found in FCs and suggested to be causal [11]. Microbial activity has also been brought in context with the potential formation of FCs [12]. Finally, the occurrence of triterpenoids, potentially resulting from *Euphorbia gummifera*, has been found in FCs of southern Namibia near Garub and suggested to be toxic for plant growth [13], a theory that has now been extended to other Euphorbia species like *E. damarana* in other regions of Namibia [14]. Given the wealth of different hypotheses, Getzin et al. [2, 15, 16] have emphasized the spatial patterns, asking which of the causal agents is able to produce the extraordinary regularity of FCs? They identified vegetation self-organization induced by strong plant competition for water to be the most plausible working hypothesis, as neither abiotic gas nor social insects in water-limited and resource-poor drylands can produce such spatially periodic patterns, and as this explanation accounts not only for observations on the Namibian FCs [5, 17, 18] but also for an identical FC pattern in Western Australia [19–21].

However, here our goal is a thorough testing of the Euphorbia hypothesis by Theron [6]. We locate several of Theron’s original metal pins with which he marked fairy circles and dead Euphorbias in the southern Giribes so long ago, which is a unique opportunity for an evaluation of this long-standing hypothesis on the cause of FCs. Obviously, Theron’s Euphorbia hypothesis can only be valid in areas with *Euphorbia damarana* because this is the shrub species he addressed in his early paper. For this reason, we primarily focus with our study on the two regions Giribes and Brandberg where both, FCs and *E. damarana*, may co-occur in some isolated and marginal areas. These areas with overlap are small because *E. damarana* is a shrub that is mostly found on rocky terrain and hill slopes but not on sandy soils where FCs predominate [22]. Given that Meyer et al. [13] extended the hypothesis of Theron to the shrub species *E. gummifera* and co-occurring FCs at Garub, we also include this region in southern Namibia into our evaluation (cf. Additional file 1: Fig. S1). The FCs at Garub, however, are very atypical. For example, they partly consist of convex heaps of sand and they have a lower soil-moisture content than the matrix [3]. Nonetheless, to better understand the fairy-circle phenomenon, we include this region into our final analysis on spatial patterns. This enables us also to put into perspective the results of Meyer et al. [14], who present alleged support for the hypothesis of Theron for *E. damarana* in the Giribes and of Meyer et al. [13], who proposed a causal link between *E. gummifera* and FCs at Garub.

Both Theron [6] and Meyer et al. [14] suggested that the dead and decaying Euphorbias are the causal agents of FCs. In contrast to the joint analysis of all live and senescent Euphorbias lumped together [14], we show size comparisons between FCs and dead Euphorbias only. This is because during our field observations we found that small Euphorbias are typically young and vital while the Euphorbias are typically dying when they reach large sizes as mature individuals. Consequently, small and young Euphorbias cause noise to size comparisons with fairy circles and diminish the power to infer causality.

Meyer et al. [14] also proposed a link between the decay of *E. damarana* and FC emergence by writing about the original study plot of Theron: “Some of these dead and decaying *E. damarana* sites were marked during 1978 […] and were still not covered by grasses in 2016”. However, they do not provide visual data evidence for this claim. Also, Theron put metal pins in four different types of locations: in fairy circles, at dead *Euphorbia damarana*, at control points in the matrix and at dead branches of Euphorbias that Theron had moved towards the matrix to determine if they alone could cause FCs. While Meyer states about these locations that “These were not carefully documented (of course no GPS’s were available then) and therefore we are not sure which pins represent dead *Euphorbias* or only *Euphorbia* branches that were moved” (M. Meyer, pers. com., June 4, 2020), we aim at identifying the positions of those remaining metal pins that can be still found at Theron’s study site. In sum, the goals of the study were as follows:

The first goal of our study was to unambiguously identify the still existing metal pins of Theron [6] in the southern Giribes using detailed ground observations, high-resolution drone imagery, as well as historical Google satellite image comparisons. We then documented how these marked sites of dead Euphorbias, FCs and control points in the matrix developed over the past four decades.

The second goal was to establish a potential size-relationship between FCs and dead *Euphorbia damarana* plants, i.e. the species for which Theron [6] formulated his Euphorbia hypothesis. To this end, we explicitly measured the canopy diameters of in total 60 dead *Euphorbia damarana* plants and compared them to the sizes of FCs. This was done in the Giribes as well as near Brandberg, where *E. damarana* and FCs may also locally co-occur. This analysis is important because according to the Euphorbia hypothesis only the dead and decaying shrubs are thought to induce a fairy circle via allelopathic interaction, but size distributions for FCs and dead shrubs have never been addressed previously.

The third goal was a thorough test of the Euphorbia hypothesis via spatial pattern analysis. We used drone mapping of plots at least 25 ha in size where FCs and Euphorbias co-occur and where their spatial patterns are thus affected by identical habitat conditions (irrespective of potential interaction between FCs and Euphorbias). A direct comparison of these patterns within the same plots is the most appropriate way for pattern-process inference and to evaluate the likelihood that Euphorbias could or could not cause the FCs. This latter analysis was done for *E. damarana* and FCs in the Giribes and near Brandberg, and for *E. gummifera* and FCs near Garub in southern Namibia (Fig. 1, Additional file 1: Fig. S1).

In a final analysis we measured the water infiltration inside FCs and outside in the matrix to determine if there are systematic differences or similarities across the three regions [5, 14, 19]. In the following, we present our findings and thereafter we discuss the observations.

## Results

### Revisiting marked Euphorbia damarana after more than 40 years

**Fig. 2:**
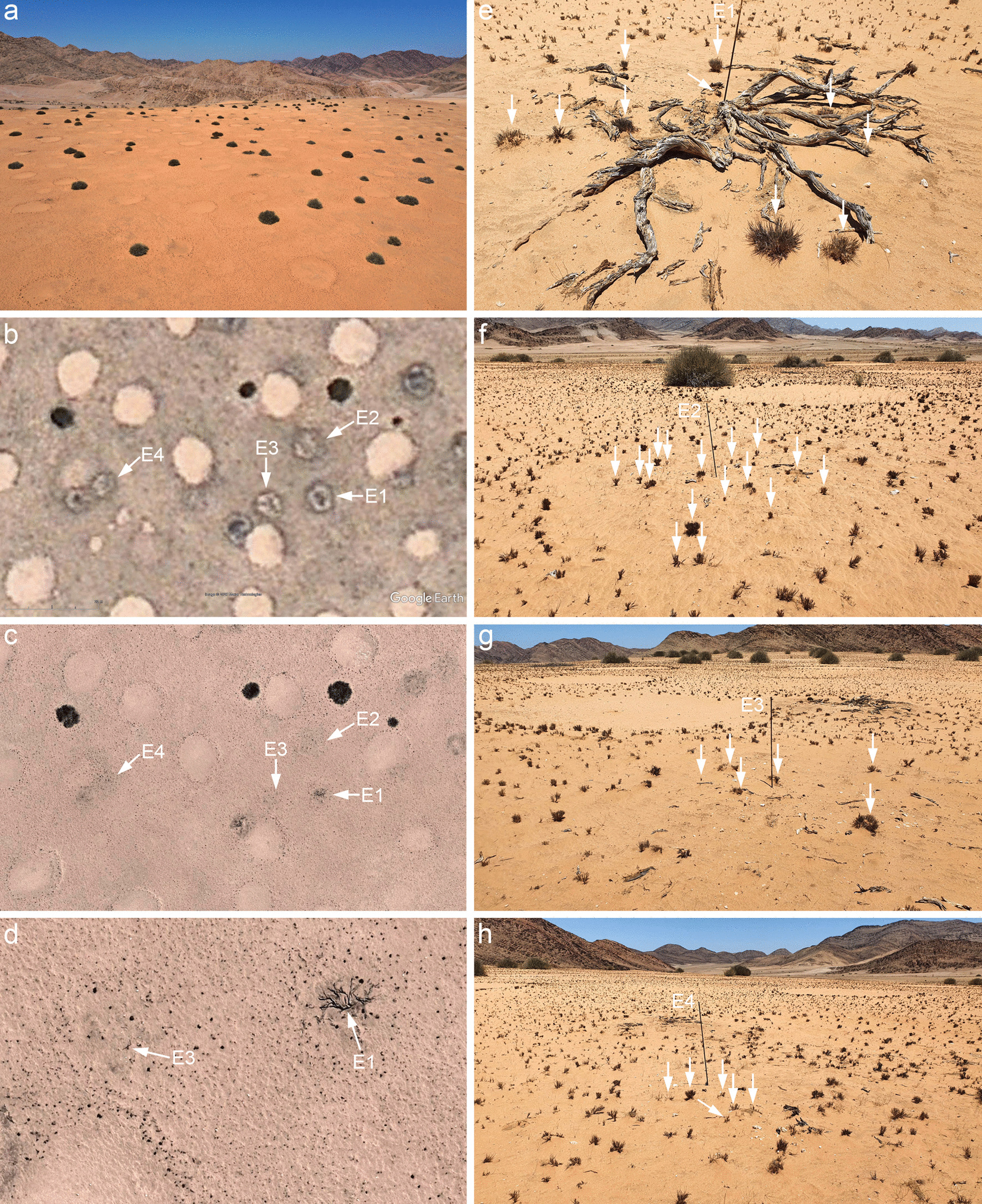
The original study site of Theron [6] in the far south of the Giribes Plains. In this plot “Gir-1” Theron marked several *Euphorbia damarana* with metal pins. Drone image taken at the site which is situated on a plateau (**a**). In this plot *E. damarana* is the only dominating shrub species and FCs are very large. Genuine fairy circles are characterized by having no vegetation growth inside the circle, as the Google satellite image from July 2009 shows (**b**). By contrast, decaying Euphorbias are characterized by dark and smaller circles where the dead branches of the shrubs lead to filled circles that clearly differ from the bright vegetation-free FCs. The arrows in the 2009 satellite image point to ground-truthed and GPS-mapped locations of four metal pins that Theron inserted at the four Euphorbias E1-E4. A drone image from March 2020 of the same marked Euphorbias (**c**). Despite the decay of the shrubs, none of the four marked Euphorbia locations developed into a fairy circle without vegetation. Also, the peripheral ring of larger grasses around the shrubs disappeared between 2009 and 2020, while the fairy circles still have this distinct ring (**c**). Zoomed drone image of the marked Euphorbias E1 and E3, with arrows pointing to the metal pins and their visible shadows (**d**). Already this drone image shows dark grass tufts growing at the metal pins of decaying Euphorbias. Ground photographs of the four marked shrubs show clearly with arrows that many large grass tufts were growing directly in the center of former Euphorbia locations and that the shrub remains had thus no phytotoxic effect (**e**–**h**). All four locations had various amounts of dead Euphorbia branches concentrated on the soil surface around the metal pins, confirming the former presence of Euphorbias and alike satellite image interpretation from 2009 (**b**)

**Fig. 3:**
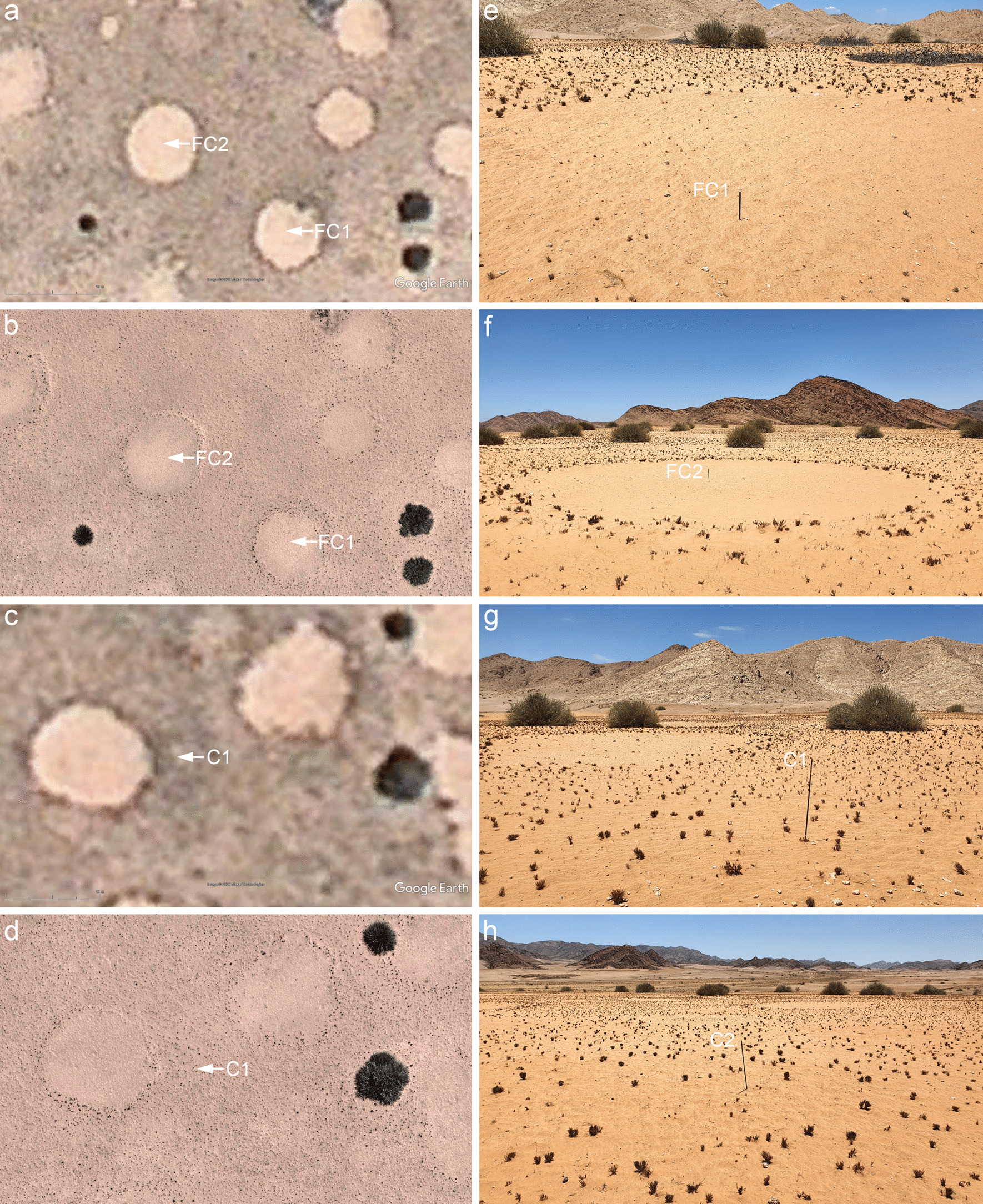
Theron [6] placed metal pins also inside fairy circles and control points in the matrix. A Google satellite image of two marked FCs from July 2009, with arrows pointing to the locations of the metal pins (**a**). A drone image of the same location, taken in 2020 (**b**). The FCs have not changed their structure over time. Note the much larger size of FCs as compared to the Euphorbias. FC1 and FC2 have diameters of 12.5 m and 13.3 m, respectively, while the two Euphorbias in the lower right corner are only half that size with diameters being 5.7 m and 5.9 m (**b**). A Google satellite image from 2009, with the arrow pointing to the marked control point in the matrix (**c**) and drone image from 2020 of the same control point (**d**). Ground images of the two marked fairy circles FC1 and FC2 (**e**, **f**). Note the absence of large grass tufts around the metal pins. Ground image of the marked control point C1 in the matrix with some stones in the foreground (**g**). Unlike for the four marked Euphorbia locations, control points had no remains of decaying Euphorbia branches around the metal pins, agreeing with a similar absence of Euphorbia structures in old satellite images from 2009 (**c**) or drone imagery from 2020 (**d**). However, control points in the matrix had the same density of larger grass tufts that can be also found under marked, decaying Euphorbias. Another example of a control point in the matrix, which also showed no Euphorbia remains on the ground but only the growth of grasses at low densities that are typical for the matrix vegetation (**h**)

In total, we found nine metal pins at the site “Gir-1” in the southern Giribes that were placed by Theron [6] more than 40 years ago (Additional file 1: Fig. S2). We identified them as four marked *Euphorbia damarana*, two fairy circles and three control points in the matrix (Figs. 2, 3). Branches that were moved by Theron and then marked were not among the metal pins because comparison with historic satellite imagery shows typical growth locations of shrubs or clear FC locations, while at the control points, we did not find any branches on the ground.

All four Euphorbia sites showed remains of dead Euphorbia branches directly around the metal pins. The Euphorbias were in different stages of decay with the shrub E1 being least decayed while the three other shrubs E2–E4 where already strongly decayed. The Euphorbia sites not only showed dead branches on the soil surface but they were additionally identified as such based on comparison between satellite imagery from 2009 and drone imagery from 2020, revealing the typical dark circle of grass tufts and blackish color within the circle that are the branches of dead Euphorbias (Fig. 2b–d). These four locations of dead *E. damarana* did not develop into FCs, as the remote sensing imagery confirms. They all showed many grass tufts growing directly at and around the metal pins and also under and in-between the branches of dead Euphorbias (Fig. 2e–h). The dying Euphorbias therefore did not prevent the growth of grasses, as suggested by the Euphorbia hypothesis.

Likewise, the two marked FCs and the three control points in the matrix were similarly identified. The two FCs did not change over time and they had no grass tufts around the metal pins (Fig. 3a, b, e, f). By contrast, the control points in the matrix all showed grasses growing directly around the metal pins (Fig. 3c, d, g, h).

### Size comparison of dead E. damarana and fairy circles

**Fig. 4:**
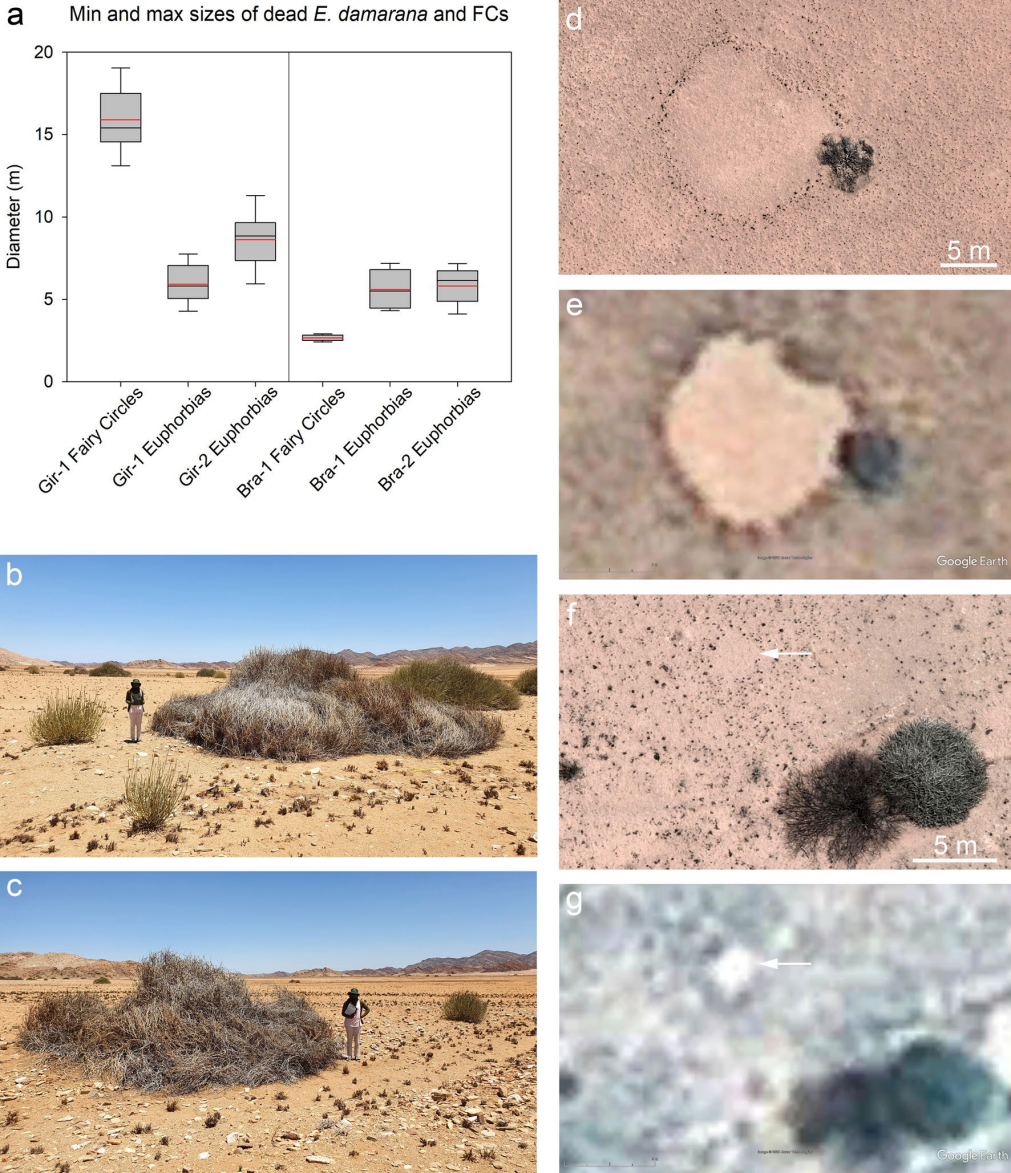
Examples of significant size differences between dead *Euphorbia damarana* and fairy circles. In the Giribes, none of the largest found dead *E. damarana* reached nearly the size of the large mega circles, which had a mean diameter of 16 m (**a**). Likewise, none of the smallest found dead *E. damarana* at Brandberg reached the size of small FCs with diameters between 2 and 3 m (**a**). In the box plots, the mean and median are indicated by the red and black horizontal lines, respectively. *Euphorbia damarana* plants only die when they have large sizes, hence small vital shrubs are irrelevant for the causal relationship as proposed by the Euphorbia hypothesis. The examples from the plot Gir- 2 in (**b**) and (**c**) show dead shrubs with an extent of 9.8 m and 7.9 m, respectively, while vital green shrubs growing next to them can be much smaller with diameters of only 1–2 m. Drone image from 2020 of a large mega circle in plot Gir-1, stretching over 16 m in extent, while the dead *E. damarana* next to it measures only 5.5 m (**d**). The sizes of the FC and shrub are stable, as the Google satellite image from 2009 shows (**e**). Drone image of a very small FC (arrow) in plot Bra-1 with a diameter of only 2.6 m, while the dead *E. damarana* in the lower right of the image has a typical diameter of 7.1 m (**f**). The small FC was already there in 2010, as the Google satellite image shows (**g**). Such small FCs cannot be caused by the typically large dead Euphorbias

Given that the Euphorbia hypothesis proposes that FCs may be caused by an allelopathic interaction between the dead shrubs and the grasses, we ground-mapped the size distribution of such dead *E. damarana* and compared it to typical FC sizes that can be found in the same study plots in the southern Giribes, as well as near Brandberg. For the southern Giribes and the plot “Gir-1”, we focused on the very large fairy circles because these were a very conspicuous landscape feature. The ten measured FCs had min, max and mean diameters of 13.0, 19.1 and 15.9 m, respectively. The ten largest found dead Euphorbias in the same 25-ha plot had min, max and mean diameters of 4.2, 7.8 and 5.9 m, respectively (Fig. 4a). In the Gir-2 plot we mapped additionally 30 dead *E. damarana*, which had min, max and mean diameters of 4.2, 11.7 and 8.6 m, respectively (Fig. 4b, c). Using one-sided *t*-tests, we found that the large fairy circles in the plot Gir-1 were at p < 0.001 significantly larger than the 40 dead Euphorbias in the plots Gir-1 and Gir-2. The largest found dead *E. damarana* did with a size of 11.8 m not even reach the size of 13.0 m of the smallest mega circle. An example of the mismatch in size of dead *E. damarana* and FCs is given for the Giribes in Fig. 4d, e.

For the Brandberg region and the plot Bra-1, we exemplarily focused only on the small fairy circles because these were a prominent characteristic of that plot. Ten of these measured small FCs had min, max and mean diameters of 2.4, 2.9 and 2.7 m, respectively. The ten dead Euphorbias in the same 25-ha plot, including the smallest dead shrubs that were present, had min, max, and mean diameters of 4.3, 7.2 and 5.6 m, respectively (Fig. 4a). In the Bra-2 plot we mapped additionally ten dead *E. damarana* in the same way, which had min, max and mean diameters of 4.1, 7.2 and 5.8 m, respectively. Using one-sided *t*-tests, we found that the small fairy circles in the Bra-1 plot were at p < 0.001 significantly smaller than the smallest found dead Euphorbias in the plots Bra-1 and Bra-2. Altogether, the smallest found dead *E. damarana* in both regions—40 shrubs in the Giribes and 20 shrubs at Brandberg—had very similar minimum diameters of about 4.1–4.3 m and can therefore not cause small FCs whose size is < 3 m. An example of the mismatch in size of dead *E. damarana* and FCs is given for the Brandberg in Fig. 4f, g.

### Comparison of spatial patterns of fairy circles and Euphorbias

**Fig. 5:**
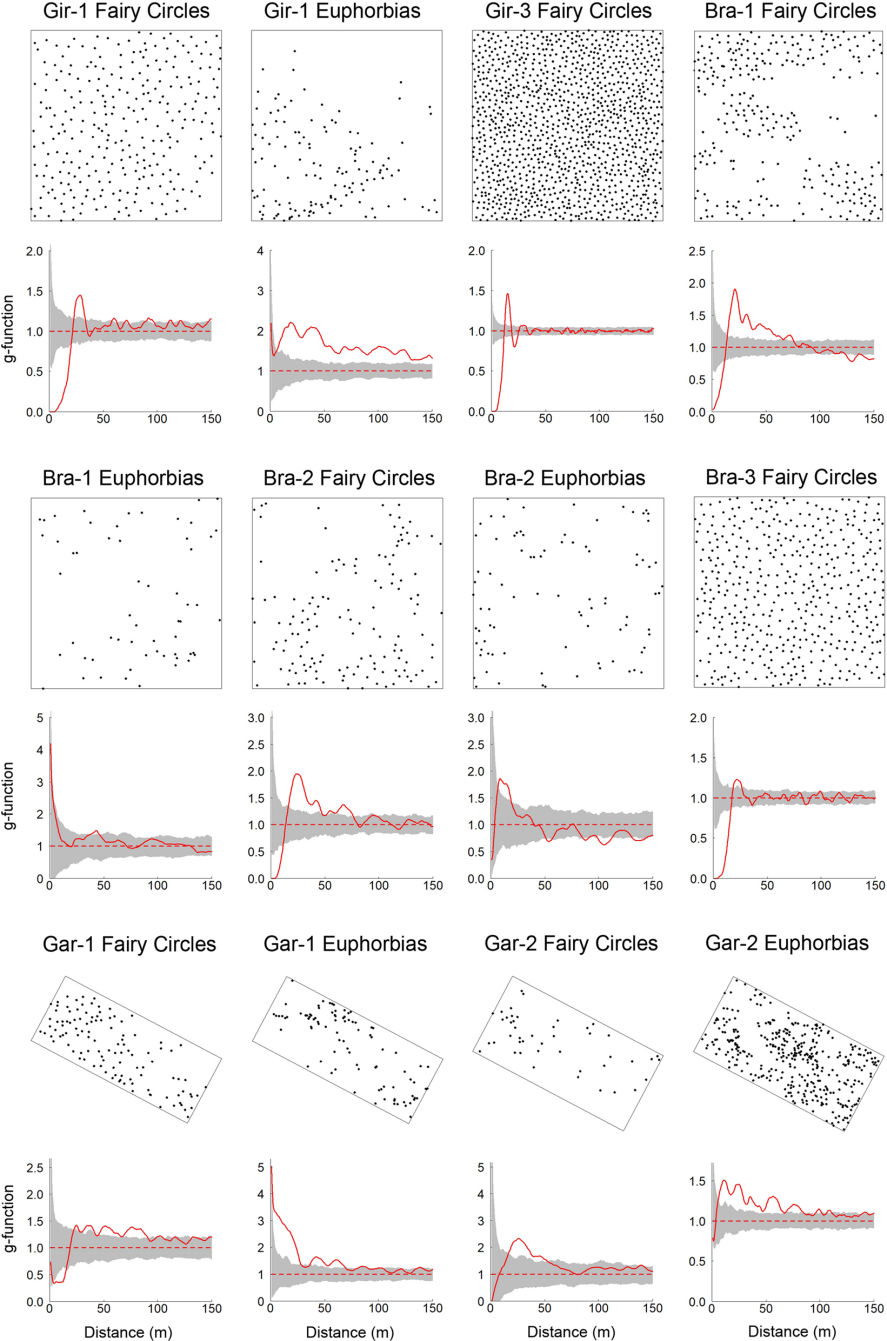
Differences between spatial distributions of fairy circles and Euphorbias, assessed with the *g*-function. The pattern is regular or aggregated at circular neighborhood distances if the red line of the *g*-function is either below the lower or above the upper grey lines of the simulation envelopes, respectively. Strong and significant fluctuations of the *g*-function far above and below the null-model envelopes, as shown for Gir-3, indicate a spatially periodic pattern. Null model envelopes were constructed using the 5th‐lowest and 5th‐highest value of 199 Monte Carlo simulations of the randomly distributed Poisson point process. The spatial patterns of the fairy circles and Euphorbias in the drone-mapped plots are shown above the *g*-functions for the Giribes (Gir-1, Gir-3), the Brandberg (Bra-1, Bra-2, Bra-3) and Garub (Gar-1, Gar-2). The *g*-function, also known as pair-correlation function, is a non-cumulative neighborhood-density function (see “Methods”)

The assessment of the spatial patterns of fairy circles and Euphorbias revealed with both, nearest-neighbor statistics and with scale-dependent correlation functions, strong differences in the distributions for the three study regions Giribes, Brandberg and Garub. In the original Theron-plot Gir-1 of the Giribes, a high Clark-Evans index *R* of 1.51 indicated strong and significant regularity for the distribution of FCs. By contrast, the *E. damarana* shrubs in the same study plot had with *R* = 0.90 an opposite pattern, being significantly clustered (Table 1). Under more homogeneous habitat conditions in the typically widely open Giribes Plains without Euphorbias nearby, represented by plot Gir-3, FCs had a very high *R*-index of 1.62, indicating very strong regularity. In this plot, the coefficient of variation (CV) of the nearest-neighbor distances was with 18.8 very low. By contrast, the high spatial variability of the Euphorbias in the Giribes plot Gir-1 is indicated by a CV of 60.7 (Table 1). Also, the *g*-functions showed regularly distributed FCs for the two Giribes plots Gir-1 and Gir-3. More specifically, for the FC-plot Gir-3 in the northern part of the Giribes Plains, the extraordinary degree of regularity is indicated by a *g*-function that strongly and significantly fluctuates around the lower and upper simulation envelopes of the random null model, hence revealing a spatially periodic pattern (Fig. 5). By contrast, the Euphorbias were primarily clustered within the plot Gir-1. This strong clustering is also evident from the *L*-function, where the red line strongly overshoots the upper simulation envelope (Additional file 1: Fig. S3).

**Table 1:** Nearest-neighbor based statistics and pattern analysis of fairy circles and Euphorbias in the three regions Giribes, Brandberg and Garub

Region	Data name	Number of points	Mean NN distance (m)	CV of NN distance	Clark-Evans index *R*	*p*-value	General type of pattern
Giribes	Gir-1 Fairy Circles	277	22.6	22.4	1.51	0.002	Regular
Giribes	Gir-1 Euphorbias	130	19.8	60.7	0.90	0.006	Clustered
Giribes	Gir-3 Fairy Circles	1143	12.0	18.8	1.62	0.002	Regular
Brandberg	Bra-1 Fairy Circles	284	17.4	42.7	1.17	0.002	Regular
Brandberg	Bra-1 Euphorbias	58	30.1	56.4	0.92	0.066	Random
Brandberg	Bra-2 Fairy Circles	138	24.2	51.4	1.14	0.036	Regular
Brandberg	Bra-2 Euphorbias	87	24.2	65.2	0.90	0.024	Clustered
Brandberg	Bra-3 Fairy Circles	437	18.0	21.1	1.50	0.002	Regular
Garub	Gar-1 Fairy Circles	113	26.6	38.9	1.10	0.32	Random
Garub	Gar-1 Euphorbias	90	21.6	95.3	0.80	0.002	Clustered
Garub	Gar-2 Fairy Circles	45	38.5	55.5	0.91	0.074	Random
Garub	Gar-2 Euphorbias	358	14.5	52.0	0.97	0.082	Random

NN = nearest neighbor, CV = coefficient of variation. Assessment of the spatial pattern was based on the Clark-Evans index *R*

Significant deviations from random distributions towards clustered or regular patterns are indicated by *p*-values < 0.05. Note that there were no Euphorbias in Gir-3 and only nine Euphorbias were in Bra-3, hence these plots have not been analyzed for Euphorbias. The specific type of pattern of the Gir-3 plot was spatially periodic (cf. *g*-function in Fig. 5), which is a special form of a regular pattern with an extraordinary degree of ordering

Similar results were also found for the Brandberg region. FCs in the 25-ha plots at Brandberg were significantly regularly distributed while *Euphorbia damarana* shrubs within the same study plots Bra-1 and Bra-2 showed either random or clustered patterns with *R*-indices of 0.92 and 0.90, respectively. The random Euphorbia pattern in the plot Bra-1 was with *R* = 0.92 and a *p*-value = 0.066 almost, but not significantly, clustering (Table 1). This is also indicated by the *g*-function, showing very high values of *g* = 4 for the first neighborhood distances (Fig. 5). By contrast, FCs had *g-*values = 0 for the first neighborhood distances, indicating that strongly competitive processes must have caused this regular pattern.

The typical clustering of regenerating Euphorbia shrubs is also evident for *E. gummifera* in the Garub plot Gar-1. A very low *R*-index of 0.80 and very high *g*-function values of *g* = 5 indicate very strong clustering of the Euphorbias within the same study plot where fairy circles occur. By contrast, the FCs in this plot Gar-1 had an *R*-index of 1.10 and their overall pattern was random, based on this nearest-neighbor index (Table 1). Still, with a *g*-function showing significant regularity of FCs for small neighborhood radii of 3–15 m, the FCs in the plot Gar-1 showed an opposite pattern as the Euphorbias within the same study plot (Fig. 5). Only in the plot Gar-2, FCs and *E. gummifera* showed similar random-dominated patterns, based on the *R*-index and the *g*-function.

In summary, in four of five study plots where FCs and Euphorbias coexist in Namibia, their spatial patterns differed significantly and the coefficient of variation in the distribution of Euphorbias was always greater than the CV of the fairy circles, indicating less ordering of the shrubs as compared to FCs.

### Infiltration measurements in fairy circles and matrix vegetation

**Fig. 6:**
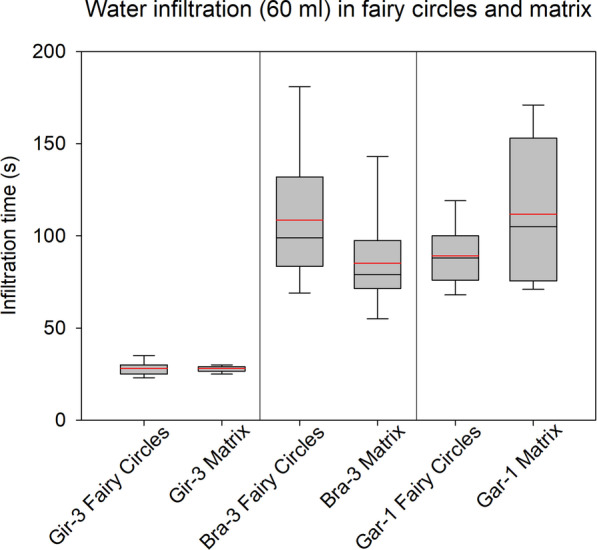
The time of water infiltration as measured for the inside and outside of fairy circles, shown for the three regions Giribes, Brandberg and Garub. In the box plots, the mean and median are indicated by the red and black horizontal lines, respectively

Our infiltration measurements revealed two main findings. First, there was no consistent difference in the rate of water infiltration for FCs and the outside vegetation in the matrix (Fig. 6). While at Garub the mean time of infiltration of 60 ml water was 89 s inside the FC and 112 s in the matrix, at Brandberg there was an opposite relation with slower mean infiltration inside the FC (109 s) but faster infiltration outside (85 s). Second, for the FC-benchmark plot Gir-3 on deep aeolian sand, water infiltration was not only exactly equal (28 s inside and outside of FC) but the speed of infiltration was also about 3.5 times faster than in the other two regions. Differences in the rate of water infiltration can therefore neither explain the absence of grasses inside the FCs on this representative reference plot in the Giribes, nor at the FC reference plot Bra-3 near Brandberg.

## Discussion

### Revisiting marked Euphorbia damarana after more than 40 years

Theron [6] marked several *E. damarana* in the southern Giribes, as well as genuine fairy circles and also locations in the matrix, that functioned as control points being away from Euphorbia shrubs or FCs. This long-term monitoring is a great opportunity to assess Theron’s early hypothesis on the origin of fairy circles.

Here, we provide for the first time detailed photographic evidence how these sites developed over the past four decades. Based on careful ground observations together with remote-sensing analysis of historic satellite and current drone imagery, we were able to identify four locations of decaying Euphorbias that also had remains of dead branches on the soil surface. These marked Euphorbias all show identical past features in Google satellite imagery from 2009 when the shrubs were still less decayed than in 2020: (1) there is a dark ring of grasses, (2) the circles are filled with dark color which are the characteristic remains of dead branches and (3) in 2020 these decaying, circular Euphorbia shrubs have approximately the same diameter as in 2009 (Fig. 2b). Together with the ground observations on the presence of dead branches, these features identify those locations with metal pins unambiguously as decaying Euphorbia shrubs that were marked by Theron [6]. Similarly, we could clearly identify two marked FCs and three control points. Our investigations of these nine metal pins highlight two main findings.

First, all four decaying Euphorbias showed grass growth directly at and around the metal pins that mark the location of these shrubs (Fig. 2e–h). Under the least decayed Euphorbia “E1”, many large tufts of *Stipagrostis* grasses were growing directly under and in-between the branches of the dead shrub. In all four locations, these grasses were not short-term annual grasses that may seasonally appear after good rainfall and disappear again in the dry season but the grasses were long-lived tufts. Hence, the decaying shrubs had no phytotoxic effect on the grasses, which falsifies the proposed allelopathic mechanism by which dead *E. damarana* would cause fairy circles.

Second, comparison between satellite imagery from 2009 and drone imagery from 2020 reveals that no FCs have formed at the locations that show the four marked Euphorbias. Despite the strong decay of the three Euphorbias E2-E4 over this time period, circular structures that would resemble neighboring FCs in drone imagery did not form (Fig. 2b, c). In other words, while the strongly decayed Euphorbias had enough time to cause the proposed phytotoxic effect and consequently a fairy circle [6], no such causation of FCs can be seen over a time span of four decades. The same finding was already obtained by van Rooyen et al. [1] who were able to relocate all the dead *E. damarana* during a field visit 22 years after Theron marked the shrubs: “no signs could be found of new barren patches being formed at dead *E. damarana* positions”. These results and our latest evidence from drone- and ground-based imagery falsifies the hypothesis of Meyer et al. [14] that “once the plant has decayed completely, a FC is hypothesized to become visible”. Contrary to this, the marked Euphorbias had a similar amount of grasses growing at the metal pins, just as the control points had in the vegetation matrix. Also, the marked FCs remained very stable over time and no large grass tufts developed within the FCs. This demonstrates that fairy circles as barren and large circular structures are different from the comparatively small locations of decaying Euphorbias.

Concerning the growth of grasses in marked Euphorbia locations, Meyer et al. [14] claim that the dead and decaying *E. damarana* sites that were marked by Theron “were still not covered by grasses in 2016”. However, they do not provide visual evidence for this surprising statement. Moreover, the supplied reference by Schutte [23] (their Fig. 4.9B, page 120) shows with only one photograph the same marked Euphorbia “E1” that we also show here (Fig. 2d, e) and already in this photograph from 2016, large grass tufts were growing in-between the dead branches of the decaying Euphorbia.

**Fig. 7:**
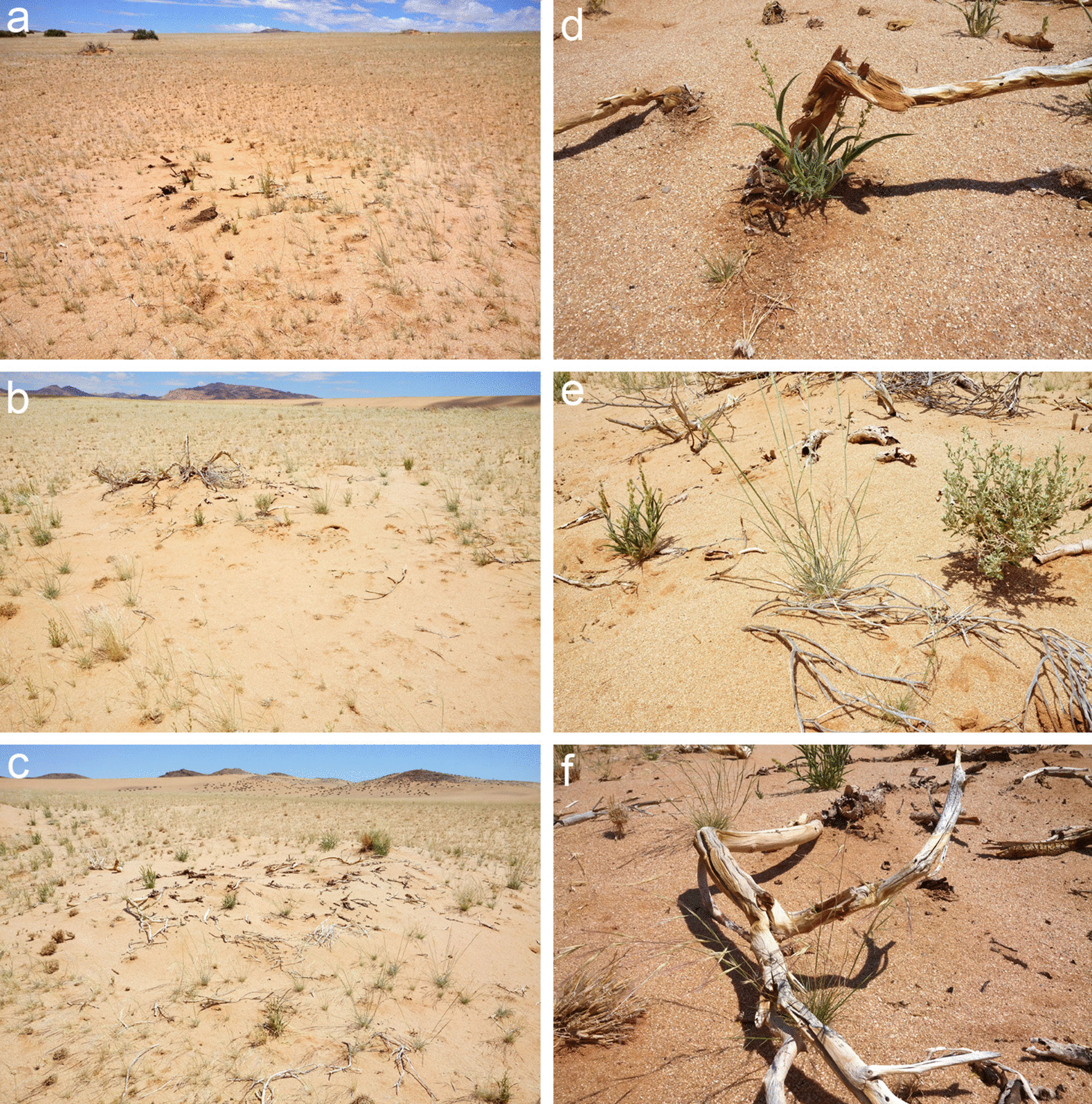
The response of grass growth to rainfall at Garub in early March 2021. Shown are three examples of decaying *E. gummifera* shrubs in the plot Gir-1 (**a**–**c**) and corresponding close-up images of the grasses growing in-between the dead Euphorbia branches (**d**–**f**). **a** Note how the grasses invade from the right side the formerly larger growth location of *E. gummifera*

The ability of grasses to grow right under decaying Euphorbia branches is also evident from the Garub site where *E. gummifera* dominates. During fieldwork in March 2021, we revisited the plot Gar-1 and found that forbs and grass genera such as *Stipagrostis* and *Schmidtia* were well thriving right at the spots where *E. gummifera* died (Fig. 7). The grasses at those locations were as vital as in the vegetation matrix and no indication of a poisonous Euphorbia effect could be seen. Generally, the fact that grasses at those shrubs did not yet have the same cover as in the matrix can be ascribed to long-term competitive effects from the shrubs and/or to depleted seed banks, as can be witnessed for many other woody plants in arid Namibia where grass growth is reduced under the canopy. However, as Fig. 7a shows, grasses do well have the ability to invade the formerly larger extent of the shrub location, thereby preventing the formation of a fairy circle.

With regard to the original study site of Theron in the southern Giribes, our visual evidence of grass growth at the marked Euphorbias agrees also strongly with previous findings from bioassays, provided by van Rooyen et al. [1]: “No inhibition of shoot or root growth was evident in the topsoil beneath either live or dead *E. damarana* plants” and “soil beneath dead *E. damarana* plants had a stimulatory effect on shoot and root growth”. This bioassay analysis revealed that *Lolium multiflorum* grasses grown in soil from under dead *E. damarana* plants had a shoot length of 253 mm, while the shoot lengths from soil of the matrix and of the barren fairy circle were only 92 mm and 61 mm, respectively. The shoot length from soil under live *E. damarana* plants was with 139 mm also very high. These significant differences in grass growth led van Rooyen et al. [1] to conclude for soil collected beneath dead *E. damarana*: “no sign of growth inhibition was found in the bioassay of this soil, rather a stimulation of plant growth was obtained and as a result the allelopathy hypothesis has to be rejected”. Also, Dean et al. [24] investigated the effects of *E. damarana* on the survival of *Aloe asperifolia* that grew directly under the Euphorbia shrubs. They made direct reference to Theron’s allelopathy hypothesis but found opposing results: “Most Aloe plants under Euphorbias were alive […] suggesting that the Aloe plants were not often destroyed by the Euphorbia”. In sum, our photographic evidence of grass growth at the marked Euphorbias supports these conclusions of van Rooyen et al. [1] and Dean et al. [24], and is in stark contrast to the Euphorbia hypothesis of Theron [6] and Meyer et al. [14]. We would like to add that G.K. Theron was author on the study of van Rooyen et al. [1], hence it is likely that he agreed with the rejection of his original hypothesis.

The large marked FCs that did not change their structure over time (Fig. 3a, b) support results by Tschinkel [25] who calculated that larger FCs have average life spans of 43–75 years. In addition, Tschinkel also provided visual data evidence based on satellite imagery from 2004 and 2008, and ground-based verifications in 2009 that new FCs develop in-between established FCs at locations where there was no Euphorbia growing before. Similar data evidence for the NamibRand Nature Reserve has been provided by Zelnik et al. [26], based on satellite images from 2004, 2008, 2010 and 2013, demonstrating that birth events occur at places where there are no Euphorbia shrubs nearby. Likewise, photographic evidence for the formation of new FCs in areas without Euphorbias has been also shown for the Marienfluss Valley [27]. Based on satellite imagery from 2004 to 2016, we provide here further evidence, even for the Theron-plot Gir-1 in the southern Giribes, that new FCs do form between established FCs at places where there was no Euphorbia shrub before (Additional file 1: Fig. S4). The dynamic formation of new FCs and their disappearance has been explained with long-term cycles of below- and above-average rainfall years [26]. In the year 2020, satellite images dating 16 years back are available and provide an excellent means to follow the temporal dynamics of both FCs and Euphorbias. Thus, we cannot agree with Meyer et al. [14] who merely speculate that new FCs in the Giribes would appear at historic growth locations of Euphorbias without providing data evidence for this claim.

In addition to historic satellite images, long-term rainfall data are an important form of real data that is available and has been shown, for example by Zelnik et al. [26], to be essential for understanding the spatio-temporal dynamics of FCs. Such data show consistently for several regions of Namibia that the majority of new born FCs appear in locations without Euphorbias. Our observation that large FCs are so stable over many decades agrees well with both, calculations on their life span [25] and with the vegetation self-organization hypothesis [26]. Model implementations of the latter show that the fixed spatial distribution of mature FCs reflects the so-called “wavelength” of the emergent pattern. The basic underlying mechanism is the landscape-scale limitation of water that equally affects all grasses within a certain area along the Namib Desert [2, 16].

### The mismatch in size of dead E. damarana and fairy circles

Also ground-based size measurements from the Giribes and the Brandberg areas show a strong mismatch between FCs and the diameter of dead *E. damarana*. According to the Euphorbia hypothesis of Theron [6] it is the hypothetical link between the dying and decomposing stages of *E. damarana* and the barren patches that may induce a fairy circle via allelopathic interaction between the dead shrubs and the grasses. He further proposed that after dying off the shrub leaves some allelopathic matter behind in the ground that could prevent the growth of grasses (his original Afrikaans writing, page 55: “Die moontlikheid bestaan dus dat *E. damarana* of enige ander diep gewortelde plantsoort met 'n groot massa bogrondse weefsel na afsterwing een of ander allelopatiese stof in die grond agterlaat wat verhoed dat ander plantsoorte, veral grassoorte wat oorwegend in die gebiede voorkom, op die kolle groei”). Also, Meyer et al. [14] collected soil from “under decaying *E. damarana* plants” to then propose that “FCs are the product of dead *Euphorbia* plants in the studied areas as indicated in the soil analysis”. Consequently, if the Euphorbia hypothesis shall be supported based on a strong similarity between the size distributions of Euphorbias and FCs, then only the size of the dying shrubs can be used to establish a potential causal link to the size of the FCs.

Neither Theron [6] nor Meyer et al. [14] have presented a statistical matching between the size of FCs and the size of only dead Euphorbias that would correspond to their own suggested causal mechanism. It is obvious that vital *E. damarana* plants grow over time from a small size of having less than 1 m diameter to mature sizes of 4, 8 or even 12 m diameter before senescence and decomposition sets in. The small and young shrubs that are green and vital, with common diameters between 1 and 3 m, will therefore grow to much larger sizes before they finally decompose. In our four study plots with measured *E. damarana* shrubs, covering a combined area of more than 100 ha, we have found smallest dead *E. damarana* with a size of about 4 m diameter. Consequently, if the many small and young vital shrubs will be included in a size comparison with FCs [14], then the size distribution of the Euphorbias is heavily biased towards small green shrubs that cannot cause a small FC because at the age of death *E. damarana* will have much larger diameters. For this reason, we have specifically measured the diameters of the dead and decaying stages of *E. damarana* and compared them to the smallest or largest diameters of the FCs that can be found in the same areas (Fig. 4). We have done this with a measuring tape on the ground because a field-based assessment of plant vitality can most accurately differentiate between green and vital Euphorbia leaves versus transitions to grey and dead vegetation. While the original Euphorbia hypothesis of Theron [6] focused with fieldwork on *E. damarana* in the Giribes area, we have extended observations on this species and on surrounding FCs also to the Brandberg region.

Any hypothesis on the origin of fairy circles must account for all characteristics of the patterning and spatial structure. A plausible hypothesis must therefore also account for observations such as finding very large or very small FC sizes in an area. We observed in two regions such extreme FC diameters with the Giribes having exceptionally large FCs while the Brandberg had many FCs with particularly small diameters. Consequently, we mapped dead Euphorbias at Giribes with a particular focus on searching for the largest possible dead *E. damarana* while at Brandberg we focused on searching for the smallest possible dead Euphorbias. We found in both regions pronounced size mismatches between FCs and dead *E. damarana*.

The Theron-Plot Gir-1 contained many very large FCs with diameters being on average 2.7 times greater than the measured dead Euphorbias in this plot (Additional file 1: Fig. S2). Ten measured FCs in this plot ranged in diameter between 13.0 and 19.1 m, while the dead *E. damarana* in the same plot ranged only between 4.2 and 7.8 m (Fig. 4). Notably, this included specifically also the largest dead shrubs that we were able to find in this 500 m × 500 m plot, but with 7.8 m the largest of all measured dead shrubs did not nearly approach the size of 13 m, which was the smallest of these ten measured mega circles. Alone in this plot there was thus no data evidence that would support the hypothesis that dying *E. damarana* could induce such very large FCs (Additional file 1: Fig. S2). Also, in the additionally mapped plot Gir-2, which is one of the rare locations in the Giribes where *E. damarana* can be found near FCs, we measured another 30 dead Euphorbias. There, we found dead shrubs ranging in size between 4.2 and 11.7 m, hence the largest dead shrub in this second plot reached also not even the smallest size of those very large mega circles that are typical for the Theron plot Gir-1. Consequently, dead *E. damarana* cannot explain the very large FCs in the Giribes.

One could still propose that horizontal transport of the allelopathic substances might over time cause the larger FC sizes. However, our data evidence from decaying *E. damarana* shrubs as well as from the large FCs (Figs. 2–4) demonstrates that the diameters did not change between 2009 and 2020, hence there was no dynamic enlargement. Moreover, if horizontal transport would enlarge the area of influence of a decaying Euphorbia, then this should always lead to an enlargement. But then the smallest dying shrubs with diameters of around 4 m would always cause much larger FCs. In this case *E. damarana* could neither explain common FC diameters of around 4 m, nor any smaller diameters from 2 to 4 m that are also common in the Gir-1 plot (Additional file 1: Fig. S2) and in the entire Giribes [15]. Irrespective of such speculations, our drone data from the Giribes show that decaying Euphorbia shrubs did not develop into FCs.

The size mismatches are also supported by our mapping of 20 dead *E. damarana* in two plots of the Brandberg region. East of Brandberg in plot Bra-1 we found very small FCs that ranged in diameter only between 2.4 and 2.9 m (Fig. 4f, g). However, in the same plot, the dead *E. damarana* ranged in size between 4.3 and 7.2 m. Another ten mapped dead Euphorbias in the plot Bra-2 north-east of the Brandberg ranged also only between 4.1 and 7.2 m. Given that it was particularly the very small FCs that were typically found at Brandberg, our mapping of dead *E. damarana* focused specifically on finding the smallest possible dead shrubs in this region. However, within the two 500 m × 500 m plots we were unable to find data evidence that dead *E. damarana* plants could induce such small FCs that are merely about 2 to 3 m in diameter. The proposed mechanism of Theron [6] and of Meyer et al. [14]—the latter authors suggested “FCs are the product of dead *Euphorbia* plants”—cannot be causal to form the observed very small FCs or the very large mega circles.

### The mismatch in spatial pattern of fairy circles and Euphorbias

Spatial statistics and pattern-process inference are one of the most powerful tools in ecology to evaluate competing hypotheses on causal mechanisms to induce certain patterns [29–31]. Ideally, patterns and potential causal agents should be compared within similar systems or habitats so that the hypothesis under investigation can be formulated as specific as possible [2]. As a consequence, we have directly compared the distribution of fairy circles and co-occurring Euphorbias within the same study plots so that FCs and Euphorbias are affected by habitat heterogeneity or other abiotic and biotic constraints in the same way. If FCs have spatial distributions that clearly differ from the distributions of Euphorbias, then the shrubs can be excluded as a potential candidate mechanism to cause the typical pattern of fairy circles [15, 28, 29].

For the Theron-plot Gir-1 where FCs and *E. damarana* co-occur, we found a distinct and significant mismatch between both patterns: FCs were strongly regular at smaller scales up to around *r* = 5 m, as indicated by the *g*-values = 0 and thus by a *g*-function that did not detect FCs at these neighborhood scales (Fig. 5). By contrast, the Euphorbia shrubs in the same plot showed random and clustered patterns at small scales. Also, the Clark-Evans *R*-indices showed a statistically significant mismatch: FCs had a high *R*-index of 1.51, indicating strong and significant regularity while the Euphorbia shrubs had a low *R*-index of only 0.90, indicating significant clustering. It is evident from these data that such significantly opposing spatial distributions cannot have the same causal mechanism.

The FCs in the Theron-plot Gir-1 did not reach the typical extreme regularity (i.e. spatial periodicity) that they attain under homogeneous habitat conditions in the wide open Giribes Plains further to the north because this plot in the far south is affected by drainage lines and other habitat heterogeneity induced by the nearby mountains. The reduced density of FCs in the south-eastern corner of the plot, that is induced by a lack of sandy substrate, resulted in an *L*-function being slightly above the upper simulation envelope of the null model (Additional file 1: Figs. S2, S3). This effect from habitat heterogeneity in the southern Giribes disrupts the spatial periodicity of the overall pattern, which can be typically found further north, as in our reference plot Gir-3 (Fig. 5). Nevertheless, FCs in Gir-1 occupied nearly the entire 25-ha plot while the distribution of Euphorbias was limited only to about half of that plot. This led to an *L*-function of the Euphorbias that strongly overshoots the upper simulation envelope and thereby indicates a strongly heterogeneous growth pattern [31]. Such large-scale heterogeneous growth patterns which indicate preferences for abiotic niches or clustered short-range dispersal are in stark contrast to the typical spatial properties of FCs in the Giribes and many other regions of Namibia, such as the Marienfluss Valley or the NamibRand Nature Reserve, where FCs are homogeneously spread over the landscape and where they attain very high *R*-indices of 1.62–1.68 and spatially periodic patterns [2, 32].

Also, in our FC-reference plot Gir-3, FCs reached the typical and unique spatial signature that distinguishes the fairy circles from other gap patterns in arid grasslands: they are spatially periodic and large-scale homogeneously distributed across the landscape. This ability to attain such a high degree of even-spaced patterning makes the fairy circles exceptional amongst all the known gap patterns in arid grasslands, hence they constitute a unique benchmark pattern against which other patterns and potential causal agents can be compared [2]. Of course, as seen in Gir-1, FCs can also show lower spatial ordering when habitat conditions are not that homogeneous (see also Cramer and Barger [17], Getzin and Yizhaq [3]). But the very high *R*-index of 1.62 for the benchmark pattern of FCs in the plot Gir-3 is higher than all *R*-indices of the Euphorbias. These unique FC patterns are therefore in stark contrast to the less ordered Euphorbia patterns because there is no data evidence that *E. damarana* shrubs could potentially create a similar strong regularity as the FC-benchmark pattern. The factual absence of such empirical data falsifies the Euphorbia hypothesis as a potential causal agent for the formation of FCs because *E. damarana* do neither create spatially periodic patterns such as in the reference FC-plot Gir-3, nor do the Euphorbias show any agreement with FC patterning within the same plot Gir-1.

The typical characteristics of spatially periodic FC patterns under homogeneous habitat conditions have been described for more than 70,000 FCs from the Giribes Plains in a very large and representative area that covers 4 km × 4 km [16]. No Euphorbias exist on these 16 km^2^ and the FCs show the identical spatial patterning as demonstrated here for the Gir-3 plot. In contrast, the increasingly narrowing mountain valley of the southern tip of the Giribes includes to an increasing extent effects from habitat heterogeneity that disrupts the degree of regularity of the FCs (Fig. 1a). For this reason, this southern area of the Giribes has been previously selected to study such effects of habitat heterogeneity on the formation of unusual FCs, such as for example, the elongated mega circles along drainage lines [3]. For their spatial point pattern analysis, Meyer et al. [14] have selected two FC plots in the southern part of the Giribes, where patterns of FCs become less regular due to this habitat heterogeneity induced by the drainage lines of the nearby mountains. For example, their plot “C1” is strongly affected by drainage lines coming from the western mountain ridge, as our field visit and also drone photo of this “C1-plot” reveals (Additional file 1: Fig. S5). This explains why their *R*-indices of FC patterns in these two plots have values of only 1.49 and 1.42.

Meyer et al. [14] lumped FC and *E. damarana* coordinates of the original Theron-plot Gir-1 (their plot named “M1”) into one data set. This cannot be explained by concerns over small sample sizes because, as our drone mapping of the Gir-1 plot demonstrates, there were 277 FCs and 130 Euphorbias in the Theron plot. This is more than enough FCs and Euphorbia plants for separate analyses of both patterns (Table 1, Additional file 1: Fig. S2). Their spatial analysis of the joint pattern indicated regularity (*R*-index of 1.36). Our separate FC and Euphorbia analyses for this plot show that their low *R*-index of 1.36 is due to a mixing of clustered Euphorbias (*R*-index of 0.90) and strongly regularly distributed FCs (*R*-index of 1.51). Given that the number of FCs in the Theron-plot was twice the number of Euphorbias, the joint pattern is dominated by the FCs. Analyzing such a mixed pattern (as done in Meyer et al. [14]) is a misleading approach in pattern-process inference because it does not reveal the true pattern of *E. damarana* or FCs, but it obliterates the significantly clumped distribution of *E. damarana*. With such an analysis, the obtained weaker regularity of the FC pattern is “fitted” to the preferred hypothesis on a Euphorbia origin but it is not an objective analysis to reveal the genuine distributions of FCs and Euphorbias, including their inherent differences.

Generally, our results from spatial comparison of *E. damarana* plants and FCs are also supported by analyses of three 25-ha plots near Brandberg. Our FC-reference plot Bra-3, which was dominated by 437 fairy circles and which only had an insufficient number of nine scattered Euphorbias, showed similar FC patterns as the Theron plot Gir-1: the *g*-function revealed very strong regularity for the first neighborhood scales and the *R*-index of 1.50 indicated a significant, strongly regular distribution. It is no surprise that this FC-reference plot at Brandberg did not reach the typical spatially periodic ordering of FCs such as in the Gir-3 plot because the environment around Namibia’s highest mountain is characterized by more topographic variation, habitat heterogeneity and a less homogeneous substrate [33].

When directly comparing the patterns of *E. damarana* and FCs within the same 25-ha plots, similar mismatching results are found for Brandberg as for the Giribes. In the plot Bra-1, FCs were significantly regularly distributed based on the *g*-function and the *R*-index but the Euphorbias showed random distributions with a tendency to clustering. Likewise, also in the plot Bra-2, FCs were significantly regularly distributed based on the *g*-function and the *R*-index. By contrast, the Euphorbias in Bra-2 showed with the *g*-function random to clustered small-scale patterns and the *R*-index showed with 0.90 a significantly clustered pattern. Hence, also at Brandberg fairy circles and Euphorbias showed significantly different patterns and consequently *E. damarana* can be excluded as a causal agent to induce the FCs.

To extend the original Euphorbia hypothesis of Theron [6] from *E. damarana* to other Euphorbias such as *E. gummifera* near Garub of southern Namibia, we have also drone-mapped the spatial patterns of two plots where *E. gummifera* and FCs co-occur. These are the same two locations in an interdune valley that have been previously investigated to reawaken the Euphorbia hypothesis after decades [13]. For the plot Gar-1, the *g*-function of the FCs indicated random patterns for the smallest scales up to *r* = 2.5 m and regularity thereafter. With 1.10, the *R*-index, however, was not significantly different from random. By contrast, the *E. gummifera* shrubs in the same plot had a strongly clustered pattern, as the high and significant deviations of the *g*-function indicate for the first neighborhood scales up to about *r* = 25 m. The *R*-index was with only 0.80 the lowest we found for all analyzed Euphorbias in this entire study, indicating strong and significant clustering of the shrubs. In the second plot Gar-2 with co-occurring *E. gummifera* shrubs and FCs, both patterns were random.

These spatial results do not support the Euphorbia hypothesis for the Garub FCs because the strong spatial clustering of *E. gummifera* plants in the plot Gar-1 contradicts the pattern we found for FCs. It needs to be highlighted, though, that many of the circles in the mixed plot with Euphorbias are not typical flat or concave fairy circles that can be found elsewhere throughout Namibia. Getzin and Yizhaq [3] emphasized that there are many “very atypical fairy circles” because soil moisture was significantly higher in the matrix than inside the FCs, which is the opposite to the typical observations elsewhere in Namibia. Likewise, they observed many such circular structures which had an atypical convex shape with remains of accumulated sand, which probably resulted from aeolian sand trapping of previously decayed Euphorbia shrubs. In Google-Earth imagery and an orthogonal bird’s-eye view, those convex piles of sand may look like a typical flat fairy circle, however, on the ground it becomes obvious that such circular piles of sand are different from genuine fairy circles (Additional file 1: Fig. S6). As highlighted previously [3], these unusual fairy circles near Garub deserve further investigations. While our spatial results do not support the Euphorbia hypothesis for the Garub site, the FCs in this region need to be more thoroughly examined. For example, it needs to be verified that circular structures as seen in satellite or aerial imagery are indeed typical flat or concave FCs but not atypical convex piles of sand. Another important question is to study the temporal dynamics of the FCs near Garub and to test if they emerge independently from nearby Euphorbias, just as they form throughout Namibia without any decaying Euphorbias in the neighborhood [25, 26]. It may be likely that near Garub typical flat FCs coexist with the unusual circular structures that may look like fairy circles only in remote sensing imagery, but are in reality atypical circular accumulations of sand that may be invaded by grasses (Fig. 7). In any case, the circles of the Garub site where *E. gummifera* co-occurs make up only a very small proportion of all fairy circles along the Namib Desert and as such cannot explain the origin of millions of FCs stretching for more than 1000 km further northwards.

With regard to the study of Meyer et al. [14], we cannot replicate how they could find regular patterns for *E. damarana* at Brandberg and for *E. gummifera* near Garub. They write “*R*-values of all sites were above 1.1 indicating overdispersion”, but their published *R*-indices for the Euphorbia-only sites “P1” and “P2” near Garub were below 1.1 and had only low values of 1.08 and 1.05 (their Additional file 2: Table S5). Notably, an *R*-index of 1 indicates a random pattern and only a significance test of this Clark-Evans statistics will reveal significant deviations towards regularity or clustering. Meyer et al. [14] do not provide such a significance test, hence the presentation of their *R*-indices does not allow conclusions on significant deviations from a random pattern. Here, we provide significance tests of the Clark-Evans indices so that deviations from 1 can be interpreted as being significantly regular or clustered. As our Table 1 demonstrates, even a relatively low index of *R* = 0.92, as well as a relatively high index of *R* = 1.10 can still be a random pattern without being significantly clustered or regular, respectively. Based on our calculated *R*-indices, the Euphorbias in the Garub plots Gar-1 and Gar-2 are clustered and randomly distributed, respectively, but we found no results that *E. gummifera* shrubs in these plots with fairy circles would be regularly spaced.

Additionally, it needs to be stressed that our spatial analysis is based on an accurate interpretation of high-resolution drone imagery, while the digitization of Euphorbia shrubs in Meyer et al. [14] is based on a coarse-grained resolution of only 0.5 m panchromatic and 1.5 m multispectral satellite images. With such relatively coarse resolutions, two nearby but separate Euphorbia shrubs that cause spatial clustering may be misclassified as one single shrub, thereby losing the clustering mechanism in plant regeneration. In Additional file 1: Fig. S7 we provide several examples for the three regions Giribes, Brandberg and Garub how *E. damarana* and *E. gummifera* shrubs typically cluster at smallest but also at larger scales. This clustering is not seen in the typical pattern of fairy circles because the FCs are separated circular structures. Our spatial statistical results revealing clustered patterns of Euphorbias agree with previous field results on the dispersal mode of the seed regenerating species *E. damarana*. Dean et al. [24] highlighted in their study “no *E. damarana* seedlings in the intershrub spaces were found growing away from stones or other plants” and they argued that “exposure to physical elements, especially extremes of temperature in the desert, may limit the growth of Euphorbia seedlings to sheltered sites under other plants”. Additionally, *E. gummifera* which prevails near Garub has been classified as a self-disperser which is a mode of short-dispersal that explains the observed clustered or random pattern [34]. It is therefore not surprising that numerous Euphorbias can be found that grow very close to nearby established plants. Finally, Meyer et al. [14] did not analyze the Euphorbia patterns within the same study plots where also FCs prevail, which would be the strongest approach for pattern-process inference. Consequently, they have not assessed how the spatial formation of FC patterns and Euphorbias evolves under identical habitat conditions where the abiotic effects on pattern formation are the same. By contrast, we demonstrate here based on the four plots Gir-1, Bra-1, Bra-2 and Gar-1 from three different regions in Namibia that FCs and Euphorbias have significantly different spatial patterns. This empirical data evidence excludes the Euphorbias to be a likely candidate mechanism to cause the fairy circles.

### The mismatch in regional and global extent of Euphorbias and fairy circles

When Theron [6] postulated his Euphorbia hypothesis, his observations were limited to isolated study sites in the far southern Giribes where *E. daramana* plants co-occur near mountain slopes with fairy circles. Likewise, Meyer et al. [14] considered for the Giribes only a very small area of 250 m × 250 m, containing both FCs and *E. damarana*. However, the typical Giribes is much larger and FCs exist without any Euphorbias in the neighborhood on an area of approximately 100 km^2^. Most of this FC area being devoid of Euphorbias exists north of the Leopard Rock (Fig. 1a)*.* This immediately leads to the question, how can hundreds of thousand FCs be caused without any Euphorbia in the neighborhood?

Becker and Getzin [9] rejected the Euphorbia hypothesis based on the fact that they “did not find any *E. damarana* specimens either in Marienfluss, the Hartmann’s Valley” or the Giribes where they worked on FCs, and they concluded that the “occurrence of *E. damarana* and fairy circles is probably of coincidental nature”. They further mentioned that, as a shrub in the Giribes Plains with FCs, only *Parkinsonia africana* may form some extensive stands. Indeed, as our field visit and drone mapping show, *P. africana* co-occurs with FCs in the north-eastern part of the Giribes on several square kilometers where it partly mixes with *Salvadora persica* on the same spot (Additional file 1: Fig. S8). However, *P. africana* is just another example of a coincidental overlap between FCs and a shrub species. Becker and Getzin [9] explained the mismatch in regional extent between FCs and *E. damarana* with different preferences for different habitats: “*E. damarana* seems to be a characteristic species of areas consisting of either very coarse material (e.g. Etendeka basalt) or having a very shallow layer of top soil” and it “almost never occurs on deep sandy soil which is a characteristic feature of areas showing fairy circles”. Also van Rooyen et al. [1] rejected the Euphorbia hypothesis by arguing “the main shortcoming of this hypothesis is that *E. damarana* prefers a stony habitat and that it seldom occurs in the sandy habitats where most of the fairy circles are found”. Few such examples where *E. damarana* occurs on deep sand can be found north of the Brandberg. However, the Brandberg area with the typical rocky terrain that characterizes the Damaraland, is a prime example for finding the majority of these shrubs growing on stony surfaces. For this reason, Mannheimer and Curtis [22] classify *E. damarana* as a shrub species that is found “mostly on rocky plains and hill slopes”. Based on these facts it can be concluded that fairy circles, for example in vast areas of the Giribes, the Hartmann’s Valley or the Marienfluss Valley cannot be caused by *E. damarana* for the trivial reason that this Euphorbia species prefers habitats that differ from most typical FC areas. This addresses also the vast areas of the Namib Sand Sea in central Namibia where FCs extend over huge areas without Euphorbias in their neighborhood.

We consider pure speculations about a potential pre-historical presence of Euphorbias in those regions as irrelevant because they are not driven by data. For example, Meyer et al. [13] speculated for the Garub region with FCs but without Euphorbias that *E. gummifera* would have previously occurred in those areas but became locally extinct under moving sand dunes in the lower lying areas. Five years later, the authors [14] speculated that climate change with increasing temperatures and drier conditions would have caused the death and complete disappearance of hundreds of thousands of Euphorbias in those areas where hundreds of thousands of FCs can nowadays be found without Euphorbias in the vicinity. They proposed that the decomposition of the dead plants would have altered the chemical properties of the sand which would now be manifested in the hydrophobicity of FC soil, but scientific data in support of such a hypothesis have not been provided for those numerous areas devoid of Euphorbias.

Generally, we cannot agree with the hydrophobicity hypothesis of Meyer et al. [14] as our measurements do not show consistent differences between water infiltration within the FCs and outside in the matrix. Their measurements of water infiltration have been done under artificial laboratory conditions, resulting in exceptionally slow infiltration times of more than 1000 s for 20 ml water. By contrast, our infiltration measurements have been done *in-situ* as in previous FC studies with a standard mini-disc infiltrometer [5, 19], and we measured an infiltration rate for 60 ml of water that was many orders of magnitude faster (e.g. 28 s). Not finding consistent results agrees with previous studies because a faster infiltration outside of FCs was also noted by Moll [8], whereas infiltration was faster inside FCs in the study by Ravi et al. [5]. But the most important finding from the Giribes was that on deep aeolian sands where tens of thousands of spatially periodic FCs but no Euphorbias occur, the water infiltration was very fast and exactly equal inside and outside of the FCs. This demonstrates that under such typical homogeneous sandy conditions, edaphic differences cannot explain the absence of grasses inside the FCs.

Finally, Meyer et al. [14] also speculate about a potential Euphorbia-related cause of the Australian fairy circles, based on the wrong assumption that hydrophobicity of the soil could be caused by previous growth of resin-containing *Triodia* grasses. They use the “soft” grass species *Triodia pungens* from the area around Newman as an example for a species whose sticky adhesive resins could possibly modify the soil properties and induce a barren fairy circle. Such speculations have to be rejected based on the fact that the Australian FCs, which occur only in a small area east of Newman, are exclusively composed of a single grass species which is *Triodia basedowii* [19, 20]. This species, however, is a so-called “hard” spinifex grass and the primary difference between the hard and soft spinifex groups relates to their ability to produce resin: the leaf epidermis of soft species contains specialized resin producing cells while hard spinifex grasses do not contain such cells and therefore are non-resinous [35]. In other words, the grass species *T. basedowii* which exclusively forms the Australian FCs cannot produce resins and induce hydrophobicity of the soil. Detailed fieldwork has shown that it is the mechanical weathering of the soil surface and related ecohydrological biomass-water feedbacks, and ecosystem-engineering of *Triodia basedowii* that is causing the Australian fairy circles [20, 21].

## Conclusion

The goal of this study was to revisit Theron’s hypothesis [6] on the origin of fairy circles after more than 40 years. We found that after four decades the growth of grasses was not hampered by the decay of *Euphorbia damarana* which rejects the hypothesis that an allelopathic interaction would cause the fairy circles. Similar results have been found for sites with *E. gummifera* at Garub. For the Giribes and Brandberg regions, we also found a strong mismatch between the size distribution of dead *E. damarana* and fairy circles which excludes the shrubs as being the cause of the circles. Also, the fact that Euphorbias had predominantly clustered and partly random distributions while FCs were regularly spaced, rejects the Euphorbias as being the cause of the fairy circles. Finally, our infiltration measurements demonstrate that edaphic properties cannot explain the absence of grasses inside FCs on deep aeolian sands of the Giribes, where hundreds of thousand fairy circles exist without any Euphorbias nearby.

When Theron [6] published his original Euphorbia hypothesis more than four decades ago, he was a pioneer in fairy-circle research. Almost nothing was known about these circles during that time. It was only until the year 2012 that more than one paper per year was published on this topic according to the *Web of Science*. Today, there is a tremendous interest in research on fairy circles and much of this interest has arisen due to the huge controversy about their origin. While we have to reject the Euphorbia hypothesis based on our data evidence, it shall be highlighted that working with Theron’s original experimental setup and finding his metal pins after more than 40 years, was a unique opportunity to study the long-term trends in vegetation change in such remote desert environments.

## Methods

### Study sites

We selected three regions in Namibia to study the coexistence of Euphorbias with fairy circles (Fig. 1). The most important study plot and primary focus of this research was the original site in the southern Giribes where Theron [6] marked fairy circles and *Euphorbia damarana* with metal pins. This plot is called “Gir-1” and located at 19° 11′ 24″ S, 13° 17′ 50″ E (Fig. 1a). It is almost exclusively dominated by the shrub species *E. damarana* (Fig. 2a). This plot was used to assess the grass vegetation at the metal pins after more than 40 years, to measure on the ground the size distribution of dead *E. damarana* plants and FCs and to map with a drone all FCs and Euphorbias on a georeferenced ortho-image on 500 m × 500 m (25 ha). This plot was largely on sand, but in the south-eastern corner, rocky and coarse soil disrupts the cover of sand. To enlarge the sample size for measured dead *E. damarana* in the Giribes, we included an additional plot “Gir-2”, where all dead Euphorbias were measured over c. 1000 m along the eastern mountain foots. This plot, located at 19° 8′ 5″ S, 13° 18′ 50″ E, is the only site for which no ortho-image was produced because the Euphorbias were typically confined only to the edges of the mountain, hence the plot was strongly elongated (Fig. 1a). A third plot “Gir-3” was drone-mapped on 25-ha in the vast Giribes Plains further north, where no Euphorbias exist and where FCs are spatially periodic over dozens of square kilometers, as reported in previous studies [2, 15, 16]. This plot on deep sand was located at 19° 1′ 55″ S, 13° 20′ 33″ E and it served as a benchmark example, representing the unique degree of spatial regularity that is typical for Namibian FCs. Mean annual precipitation (MAP) for those plots is approximately 100 mm.

We extended the study to the Brandberg region because *E. damarana* and FCs may also there locally co-exist. The plot “Bra-1” was located east of Brandberg at 21° 2′ 58″ S, 14° 51′ 2″ E and this plot had many small FCs with diameters of 2–3 m (Fig. 1b). It was used for measuring on the ground the size of dead *E. damarana* and of FCs and for mapping on 25 ha the location of all FCs and *E. damarana* with drone imagery. The plot had several small drainage lines running through it and also Acacia reficiens was relatively abundant. Another plot with scattered boulders, “Bra-2”, was located north-east of Brandberg at 20° 58′ 6″ S, 14° 40′ 45″ E. It was used for measuring on the ground the size of dead *E. damarana* and for mapping on 25 ha all *E. damarana* and FCs. Also, this plot had other woody species such as *Boscia foetida*, *Commiphora saxicola* and *Parkinsonia africana*. Finally, a third plot “Bra-3”, located north-east of Brandberg at 20° 58′ 46″ S, 14° 40′ 24″ E, was used as a reference plot where fairy circles dominate the landscape on sand. Only drone-mapping on 500 m × 500 m was applied for this plot because the primary question was, what type of patterns do FCs form when Euphorbias are largely absent? MAP for the three plots is approximately 100 mm.

Given that the region around Garub in southern Namibia has been previously used to extend the original Euphorbia hypothesis to *E. gummifera* and FCs [13, 14], on 18th March 2020, we also drone mapped two plots there. The plots are the same two locations that have been analyzed before by Meyer et al. [13] and by Getzin and Yizhaq [3]. Both plots on sand are located within an interdune valley which restricts the study plots to a rectangular shape (Fig. 1c). The plot “Gar-1” is located at 26° 36′ 16″ S, 16° 1′ 2″ E and drone-mapping was undertaken for a size of 800 m × 330 m (26.4 ha). The plot “Gar-2” is located at 26° 37′ 20.07″ S, 16° 4′ 1.10″ E and due to a wider valley, drone-mapping was undertaken for a size of 800 m × 400 m (32 ha). MAP for this site is approximately 60 mm.

No plant samples have been collected during fieldwork and no voucher specimen have been deposited in a publicly available herbarium. The identification of the plant species has been done in the field by S. Getzin and A. Nambwandja.

### Drone mapping of sites

We used the drone *DJI Mavic 2 Pro* for mapping the fairy circles and Euphorbias with a Hasselblad photo camera. This camera with a wide-angle lens of 35 mm (28 mm equivalent at full-frame format) has a 1″ CMOS sensor and 20 MP. The drone flew at a speed of 5 m/s and the plots were mapped with 80% forward and 40% sideward overlap of images. The camera was set to an aperture priority of f/5.6 and an ISO speed of 200, allowing short shutter speeds of 1/2000 and shorter under clear skies. For each JPEG image, meta data on the flying altitude and x,y-coordinate were recorded. All images were processed in OneButton software (www.icaros.us) to stitch an orthorectified and geo-referenced aerial image that covers 500 m × 500 m (25 ha) or 26.4 and 32 ha for the rectangular plots Gar-1 and Gar-2, respectively. The final orthophotos had a resolution of 3 cm/pixel. This high image resolution allowed a precise identification of FCs and Euphorbia shrubs in the orthophotos. Identification of *E. damarana* and *E. gummifera* in our high-resolution drone imagery was easily possible because unlike other shrub and tree species, the Euphorbias are characterized by a distinct round crown shape with very tightly packed leaves. We manually delineated all visible Euphorbia shrubs in each plot, including the dying ones whose original size extent was still visible. For each segmented FC and Euphorbia we created shapefiles (a geospatial vector format, digitized with QGIS-3.10.5 software, https://qgis.org/en/site/) with geo-referenced information on the object’s x,y-coordinate.

### Revisiting marked Euphorbia damarana after more than 40 years

From 2nd to 4th March 2020, we visited several sites in the Giribes Plains, including the original study site of Theron [6] in the southern Giribes, named “Gir-1”. The goal of this field visit was to identify and photograph the metal pins that Theron inserted more than 40 years ago at locations of Euphorbias, fairy circles and control points in the matrix vegetation. Upon finding those metal pins, their positions were recorded with a hand-held GPS device and the immediate neighborhood of the pins was investigated for visible remains of dead Euphorbia branches and/or grass tufts. All nine located metal pins were photographed on the ground to provide visual data evidence for the status of those marked locations. The locations with metal pins were also photographed with a drone, *DJI Mavic 2 Pro* and the GPS information of each photo was stored as metadata for each image which enabled an exact matching of ground-based GPS recordings, drone-based imagery and the same imagery from Google satellite.

### Size comparison of dead E. damarana and fairy circles

We observed in the two regions Giribes and Brandberg FCs with very large and very small diameters, respectively. Hence, for the Giribes we were asking, if there are large dead *E. damarana* plants whose size would match the size of the very large FCs. At Brandberg the natural conditions were different and not very large but very small FCs were noticed during the survey. Consequently, we were asking if there are particularly small *E. damarana* shrubs whose small size would match the small FCs. The primary goal of this mapping was to find potential data evidence that decaying Euphorbia shrubs were able to induce such extreme FC diameters, because it is only the dead Euphorbias that are expected to cause the FCs [6, 14]. If the Euphorbia hypothesis were valid, it should be able to account for all FC observations, including the very large and small diameters.

In the Theron-plot Gir-1, we mapped the size (diameter) of ten particularly large fairy circles that co-occur with *E. damarana* in the same 25-ha study plot. A measuring tape was used to record the length of the longest side of a fairy circle in case the circle was slightly oval. In the same way, we recorded the length of the longest side of ten dead *Euphorbia damarana*, which included the largest dead shrubs that we could find on an area of 500 m × 500 m. Dead shrubs were classified as dead if they had predominantly grey leaves or if they showed already signs of decay with branches lying on the ground. By contrast, vital shrubs that we did not measure had green leaves and were standing upright without signs of decay. Given that Euphorbia shrubs are rare in the Giribes Plains and that they only occur in the far south, we additionally mapped another 30 dead *E. damarana* along the eastern mountain ridge, close to the Theron-plot Gir-1. In this plot Gir-2, we moved from north to south and recorded over a distance of circa 1 km, the size of all dead Euphorbias (Fig. 1a). The recording of these dead shrubs included the largest individuals that were present in the two plots Gir-1 and Gir-2.

Since *E. damarana* shrubs are very common around Brandberg, we also recorded the size of dead shrubs east and north-east of Brandberg, where they co-occur with FCs. This mapping was done on 28th and 29th February 2020. In the 25-ha plot Bra-1, we noticed a number of very small FCs whose diameters were below 3 m. Small FCs that range in size only between 2 and 3 m are also well known from the Giribes Plains where Euphorbias are typically absent [15], but at the Brandberg plot Bra-1, they co-occurred with *E. damarana* in the same plot. Therefore, we measured in this plot ten dead *Euphorbia damarana* and ten small FCs to compare their size distributions. Additionally, we also measured the sizes of another ten *E. damarana* in the 25-ha plot Bra-2 north-east of the Brandberg and thus north of the Ugab River. Contrary to the Giribes with very large FCs, the mapping of the dead *E. damarana* plants at Brandberg focused on including the smallest individuals that were present in the two plots Bra-1 and Bra-2.

Finally, one-sided *t*-tests were used to evaluate if the large mega circles in the Giribes were significantly larger than the recorded sizes of dead shrubs and if the small FCs at Brandberg were significantly smaller than the dead *E. damarana* shrubs.

### Comparison of spatial patterns of fairy circles and Euphorbias

We applied spatial point pattern analysis to the locations of FCs and Euphorbias in the seven drone-mapped study plots of three regions in Namibia. For the Giribes region, FCs and *E. damarana* were compared within the same study plot Gir-1. Additionally, a reference plot Gir-3 where only FCs occur was analyzed to characterize the typical, spatially periodic, pattern of FCs that can be found in many regions of Namibia. In the Brandberg region, we analyzed again the patterns of FCs and *E. damarana* within the same two study plots Bra-1 and Bra-2, and additionally, we used a reference plot Bra-3 to analyze the FC pattern where Euphorbia shrubs are largely absent. In the Garub region, we focused only on the two mixed sites with FCs and *E. gummifera*, Gar-1 and Gar-2, which have been previously used to reactivate the Euphorbia hypothesis [13].

We used two main types of spatial analysis: nearest-neighbor based statistics, namely the Clark-Evans *R*-index and the coefficient of variation (CV) of the nearest-neighbor distances and scale-dependent point pattern analysis based on the *g*-function and *L*-function [2, 30, 36].

The Clark-Evans index [37] is calculated as $R = \overline{r}_{A} /\overline{r}_{E}$ where $\overline{r}_{A}$ is the average distance from randomly selected points to their nearest neighbors and $\overline{r}_{E}$ is the theoretically expected average distance between nearest neighbors under a Poisson distribution of complete spatial randomness (CSR). *R* = 1 for random patterns, whereas clumped (aggregated) patterns have values of *R* < 1 and regular (overdispersed) patterns have values of *R* > 1. For comparative reasons, the *R*-index is presented without edge correction because uncorrected results have been primarily published in the past. In order to assess significant deviations from randomness with *R* = 1, a *p*-value for a two-sided test was computed by Monte Carlo simulation of 999 realizations of CSR conditional on the observed number of points. Additionally to the *R*-index, we also calculated the CV of the nearest-neighbor distances because a very low variation in distances is typical for the very strong spatial regularity and ordering of fairy circles [2].

To assess the scale-dependent spatial patterns of FCs and Euphorbias, we applied the *g*-function which is also called pair-correlation function. This non-cumulative neighborhood-density function *g*(*r*) is the expected density of points at a given distance *r* from an arbitrary point, divided by the intensity (*λ*) of the pattern [38, 39]. *g*(*r*) = 1 for a Poisson point process (CSR). Due to its non-cumulative approach, it is highly effective for analyzing critical scales of patterning and it is “the most powerful summary characteristic when used in isolation” [40]. Typically, the CSR null model generated by a Poisson point process is used to construct simulation envelopes to indicate deviations from *g*(*r*) = 1 as statistically significant [41]. Under regularity, values of *g*(*r*) = 0 may occur at small spatial scales and indicate that there are no points within the immediate neighborhood. Significant *g*(*r*) values outside the simulation envelopes with *g*(*r*) < 1 indicate regularity, while *g*(*r*) > 1 indicates aggregation [42]. If the *g*-function strongly fluctuates at small scales around the simulation envelopes of the null model, this indicates a spatially periodic pattern with a distinct density peak at the radius where the first six, approximately equally spaced, nearest neighbors occur [2, 16]. Unlike Voronoi tessellations which are used to merely count the mean number of nearest neighbors [15], which can be six for regular but also six for random patterns, the strong fluctuation of the *g*-function around the null model indicates a pattern where the six nearest-neighbor distances are approximately equal, leading to a spatially periodic distribution [2]. Such spatially periodic patterns are a special form of a regular distribution because they are more strongly ordered.

Additionally, to the *g*-function, we also used the *L*-transformation (*L*-function) of Ripley’s cumulative *K*-function, *L*(*r*) = (*K*(*r*)/π)^0.5^–*r*. Due to its cumulative properties, the *L*-function does more accurately assess departures from CSR at larger scales (i.e. 50—250 m) than the non-cumulative *g*-function [36]. For a random pattern, *L*(*r*) = 0 while *L*(*r*) < 0 indicates regularity and *L*(*r*) > 0 indicates aggregation. If the *L*-function, after the first small-scale deviations from CSR, moves consistently into the null-model envelopes of CSR, this indicates a pattern that is homogeneous at a large scale. In contrast, significant departures from the CSR null model at these large scales indicate that a pattern is heterogeneously distributed [31, 36]. The scale-dependent functions *g*(*r*) and *L*(*r*) of the 12 FC and Euphorbia patterns were tested against CSR using the 5th-lowest and 5th-highest values of 199 Monte Carlo simulations for constructing approximately 95% simulation envelopes of the CSR null model [36, 41]. All spatial analyses were carried out using *R*-software (package Spatstat, http://www.R-project.org/).

### Infiltration measurements in fairy circles and matrix vegetation

During the field season in 2021, we visited all study sites again. In each of the three regions Giribes, Brandberg and Garub we selected one 25-ha plot for undertaking water-infiltration measurements. We selected for each region those plots that had the highest number of FCs (Table 1) because the goal was to examine the origin and potential drivers of FCs. On 3rd April 2021 we measured the infiltration in the plot Gir-3 in the Giribes, which is our reference plot for spatially periodic FCs on deep aeolian sands, where no Euphorbias exist over vast areas. On 10th March 2021 we undertook infiltration measurements near Brandberg at the plot Bra-3 which had 437 FCs but only nine *E. damarana*. For the Garub region we used the plot Gar-1 on 3rd March 2021, which also had more FCs than Euphorbias. In each of the three study plots we randomly selected three FCs and undertook three infiltration measurements inside each FC and three measurements outside, approximately 2–3 m away from the FC peripheries. Hence in total we measured in each study plot nine times the water infiltration inside and nine times outside of the FCs. The time of 60 ml water infiltration was assessed with a mini-disc infiltrometer (www.metergroup.com) as used in previous FC research [19]. For each of the three study regions we plotted the recorded seconds of all infiltration measurements, including the means and medians (Fig. 6).

## Supplementary Information

**Additional file 1.** Google satellite image showing the three study regions Giribes and Brandberg (with *Euphorbia damarana*) and Garub (with *E. gummifera*). **Fig. S2.** Google satellite image from 2016 of the plot Gir-1, where Theron [6] marked the Euphorbias, fairy circles and control locations in the matrix. The white square shows the 500 m × 500 m extent of the drone-mapped plot. **Fig. S3.** The large-scale spatial distributions of fairy circles and Euphorbias, assessed with the *L*-function. The pattern is regular or aggregated at circular neighborhood distances if the red line of the *L*-function is below the lower or above the upper grey lines of the simulation envelopes, respectively. **Fig. S4.** Formation of new FCs in the Theron-Plot Gir-1. The four Google-Earth satellite images from 2004 to 2016 show how FCs emerge at places where there was no Euphorbia before (a-d). **Fig. S5.** This drone image shows the “C1-plot”, as used for spatial analysis of FCs by Meyer et al. [14]. This plot, however, is not representative for the spatially periodic ordering of FCs that can be seen in vast parts of the Giribes Plains further north under more homogeneous habitat conditions. **Fig. S6.** Several examples of typical sand piles, accumulating around decaying *Euphorbia gummifera* at the two sites Gar-1 and Gar-2 near Garub (a-d). These circular sand piles with reduced vegetation cover may look similar to fairy circles in aerial or satellite imagery. However, these piles of sand cannot be called fairy circles. **Fig. S7.** Evidence for clustered patterns of *E. damarana* and *E. gummifera*. *Euphorbia damarana* in the Theron-plot Gir-1 of the southern Giribes (a) and in the plot Bra-2 near Brandberg (b) showed statistically significant clustering because regeneration of the shrubs often happens in a clumped distribution where individual shrubs are tightly packed together (arrows). Also, *Euphorbia gummifera* in the plot Gar-1 near Garub showed strongly clustered patterns (c) while in the plot Gar-2 the Euphorbias showed a mixed pattern of clustered and random distributions (d). Oblique drone images with overviews over the plots Gar-1 (e) and Gar-2 (f) near Garub clearly show how the individual shrubs with their humped growth form are tightly clustering but they do not have a regularly spaced pattern. **Fig. S8.** In the north-eastern part of the Giribes Plains, the species *Parkinsonia africana* co-occurs in various densities with fairy circles over an area of several square kilometers. Two examples have been photographed in March 2020 with a drone (a, b). *Parkinsonia africana* is a shrub species or small tree, which may grow on its own (c) or it may be occupied by *Salvadora persica* which scrambles into it (d)

**Additional file 2.** The file “Additional_Spatial_Data_FCs_Euphorbias.xlsx” is an Excel spreadsheet that contains all drone-mapped x,y-coordinates of fairy circles and Euphorbias for the three regions Giribes, Brandberg, Garub, as summarized in Table 1.

## Data Availability

The data sets generated and analyzed during this study are included as Excel files in this published article and its additional information. Other raw data such as aerial imagery are confidential, as they are subject to ongoing publications and will be published at a later stage.

## Abbreviations

CSR: Complete spatial randomness
CV: Coefficient of variation
FC: Fairy circle
MAP: Mean annual precipitation
NN: Nearest neighbor
