# Supplementary material for: Revisiting Theron’s hypothesis on the origin of fairy circles after four decades: Euphorbias are not the cause
Source: BMC Ecol Evol. 2021 May 28;21:102. doi: 10.1186/s12862-021-01834-5 (PMC8162014; doi:10.1186/s12862-021-01834-5)
Supplement: Supplementary file 1 — Additional file 1. Google satellite image showing the three study regions Giribes and Brandberg (with Euphorbia damarana) and Garub (with E. gummifera). Fig. S2. Google satellite image from 2016 of the plot Gir-1, where Theron [6] marked the Euphorbias, fairy circles and control locations in the matrix. The white square shows the 500 m × 500 m extent of the drone-mapped plot. Fig. S3. The large-scale spatial distributions of fairy circles and Euphorbias, assessed with the L-function. The pattern is regular or aggregated at circular neighborhood distances if the red line of the L-function is below the lower or above the upper grey lines of the simulation envelopes, respectively. Fig. S4. Formation of new FCs in the Theron-Plot Gir-1. The four Google-Earth satellite images from 2004 to 2016 show how FCs emerge at places where there was no Euphorbia before (a-d). Fig. S5. This drone image shows the “C1-plot”, as used for spatial analysis of FCs by Meyer et al. [14]. This plot, however, is not representative for the spatially periodic ordering of FCs that can be seen in vast parts of the Giribes Plains further north under more homogeneous habitat conditions. Fig. S6. Several examples of typical sand piles, accumulating around decaying Euphorbia gummifera at the two sites Gar-1 and Gar-2 near Garub (a-d). These circular sand piles with reduced vegetation cover may look similar to fairy circles in aerial or satellite imagery. However, these piles of sand cannot be called fairy circles. Fig. S7. Evidence for clustered patterns of E. damarana and E. gummifera. Euphorbia damarana in the Theron-plot Gir-1 of the southern Giribes (a) and in the plot Bra-2 near Brandberg (b) showed statistically significant clustering because regeneration of the shrubs often happens in a clumped distribution where individual shrubs are tightly packed together (arrows). Also, Euphorbia gummifera in the plot Gar-1 near Garub showed strongly clustered patterns (c) while in the plot Gar-2 the Eupho [file 12862_2021_1834_MOESM1_ESM.docx]

Additional information for:

**Revisiting Theron’s hypothesis on the origin of fairy circles after four decades: Euphorbias are not the cause**

Stephan Getzin^1,2,*^, Ailly Nambwandja^3^, Sönke Holch^1^ and Kerstin Wiegand^1^

^1^Department of Ecosystem Modelling, University of Goettingen, Goettingen, Germany

^2^Department of Ecological Modelling, Helmholtz Centre for Environmental Research – UFZ, Leipzig, Germany

^3^Gobabeb - Namib Research Institute, Namib Naukluft Park, Namibia

*Corresponding author: stephan.getzin@uni-goettingen.de


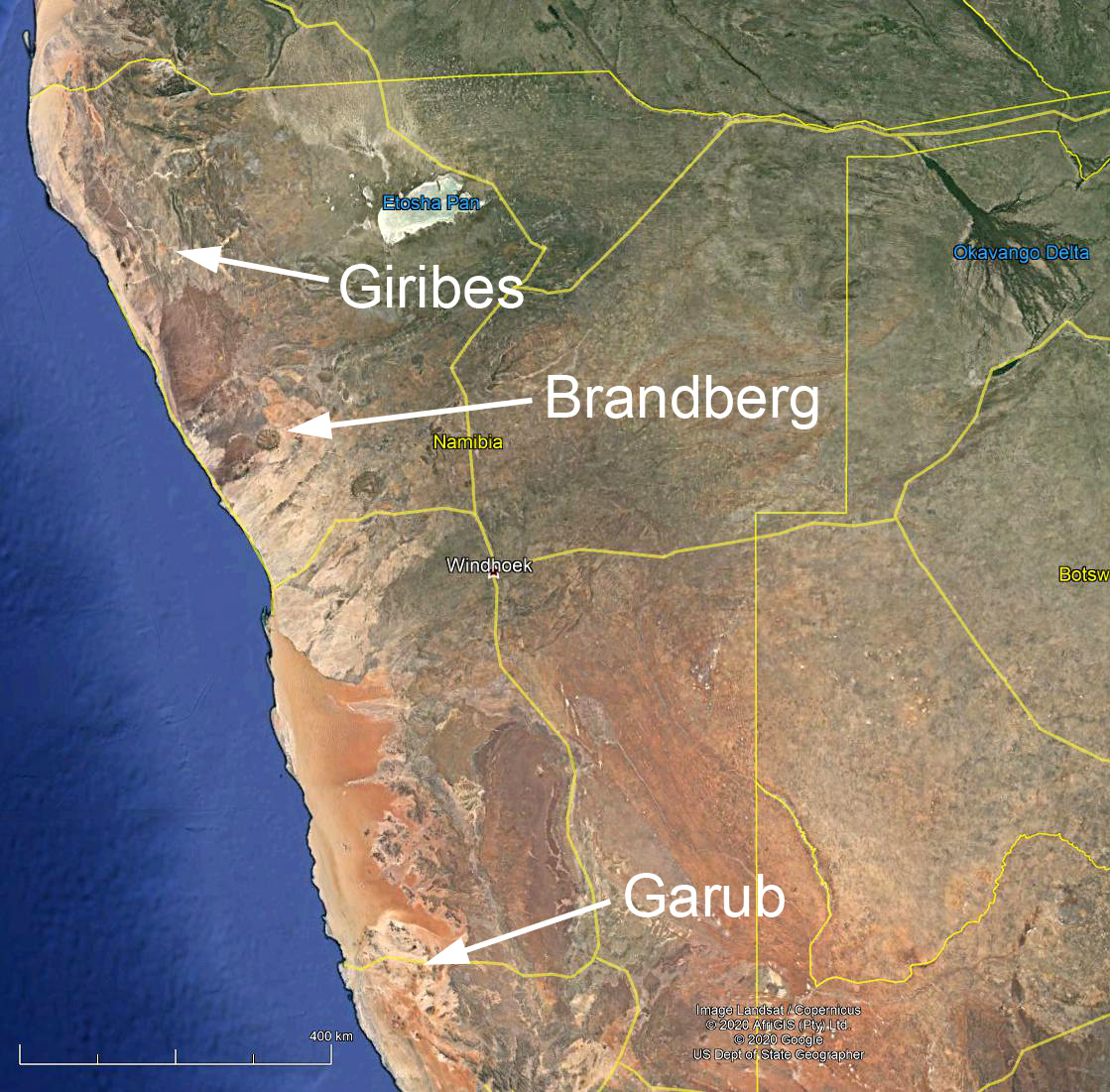


**Fig. S1** Google satellite image showing the three study regions Giribes and Brandberg (with *Euphorbia damarana*) and Garub (with *E. gummifera*).


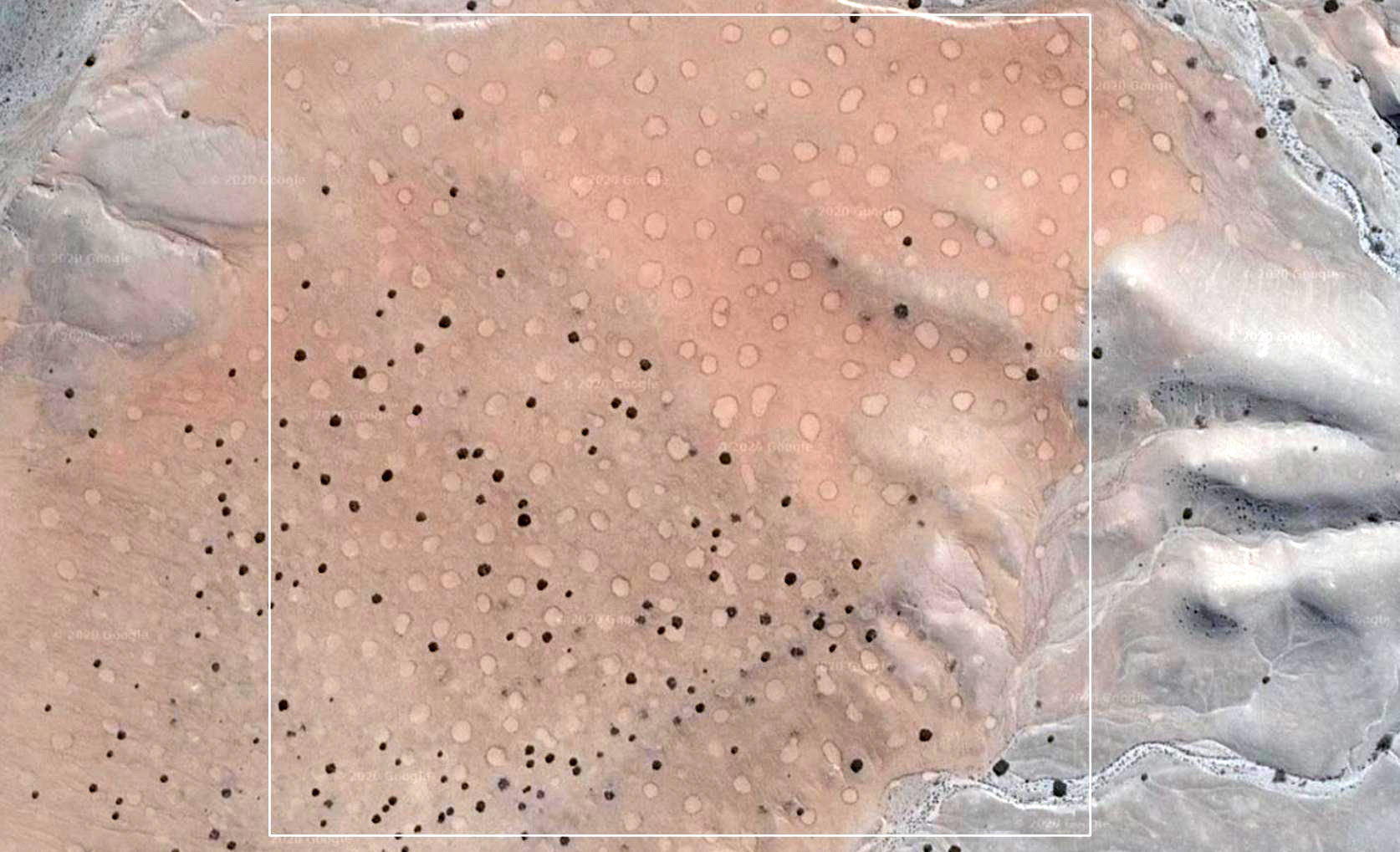


**Fig. S2** Google satellite image from 2016 of the plot Gir-1, where Theron [6] marked the Euphorbias, fairy circles and control locations in the matrix. The white square shows the 500 m × 500 m extent of the drone-mapped plot. As the image shows, there are numerous FCs and *Euphorbia damarana* to enable statistical comparisons between the two types of objects, including spatially-explicit analysis of the patterns with the *g*- and *L*-functions. Note the large sizes of the FCs while Euphorbias are comparatively small.


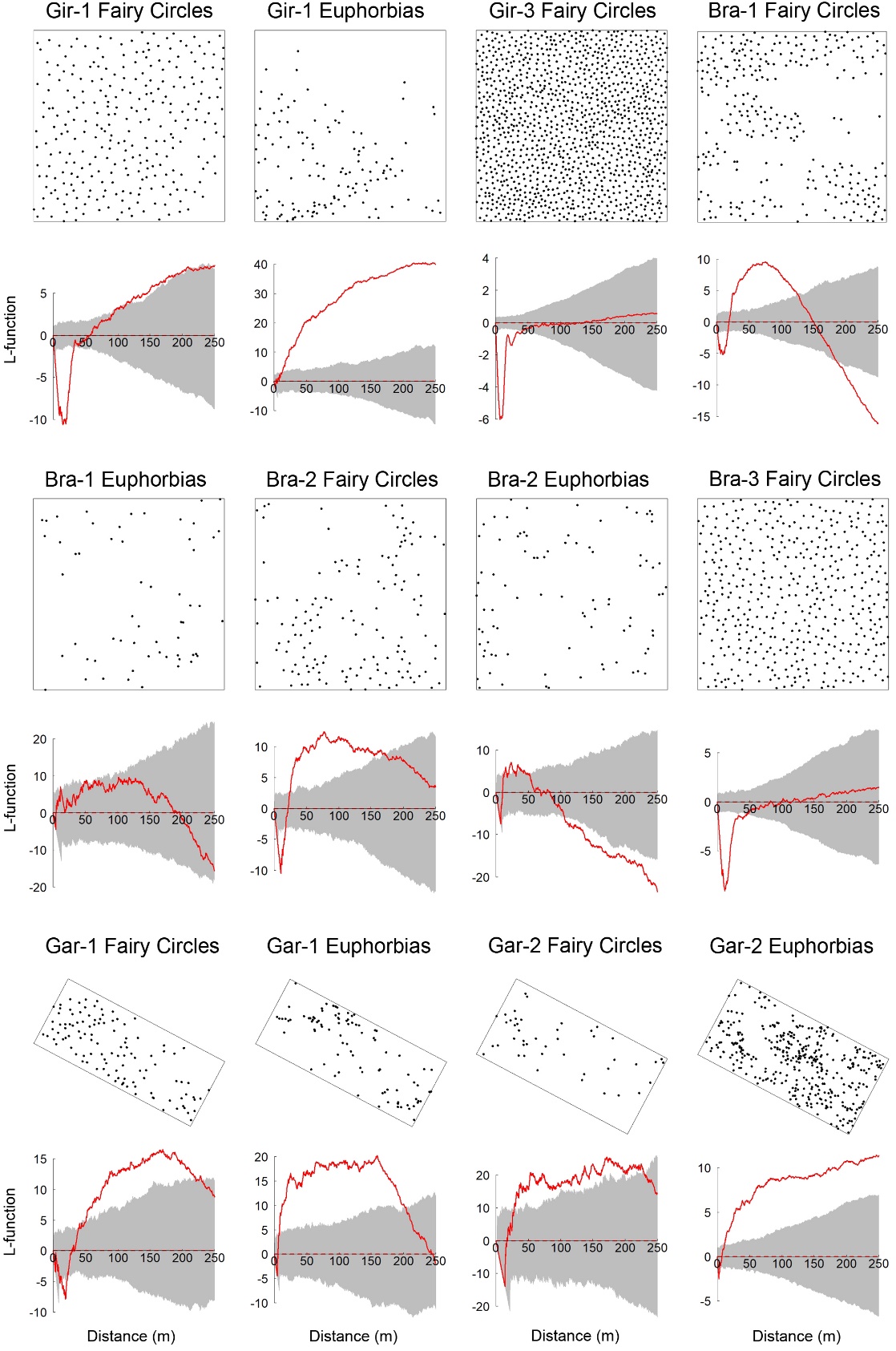


**Fig. S3** The large-scale spatial distributions of fairy circles and Euphorbias, assessed with the *L*‑function. The pattern is regular or aggregated at circular neighborhood distances if the red line of the *L*-function is below the lower or above the upper grey lines of the simulation envelopes, respectively. Null model envelopes were constructed using the 5th‐lowest and 5th‐highest value of 199 Monte Carlo simulations of the randomly distributed Poisson point process (CSR). The mapped point patterns of the FCs and Euphorbias are shown above the *L*-functions for the Giribes (Gir-1, Gir-3), the Brandberg (Bra-1, Bra-2, Bra-3) and Garub (Gar-1, Gar‑2).


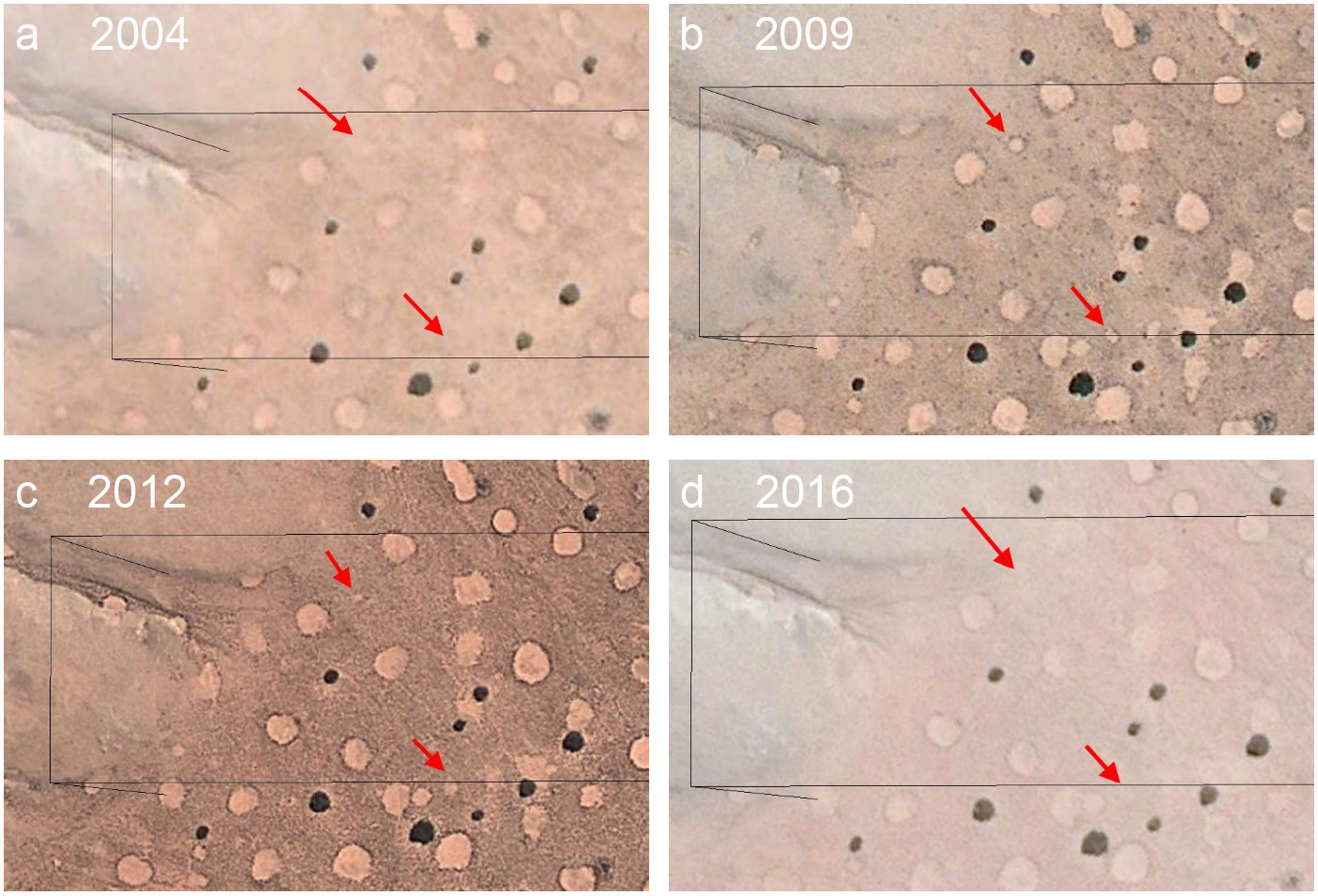


**Fig. S4** Formation of new FCs in the Theron-Plot Gir-1. The four Google-Earth satellite images from 2004 to 2016 show how FCs emerge at places where there was no Euphorbia before (a-d). The arrows in the image from 2009 point to two examples of new FCs (b), while there was no Euphorbia at that location in 2004 (a). Large, established FCs are long-lived structures with lifespans reaching at least half a century. However, new born FCs may disappear again (d), which is mainly attributed to cycles of rainfall variation.


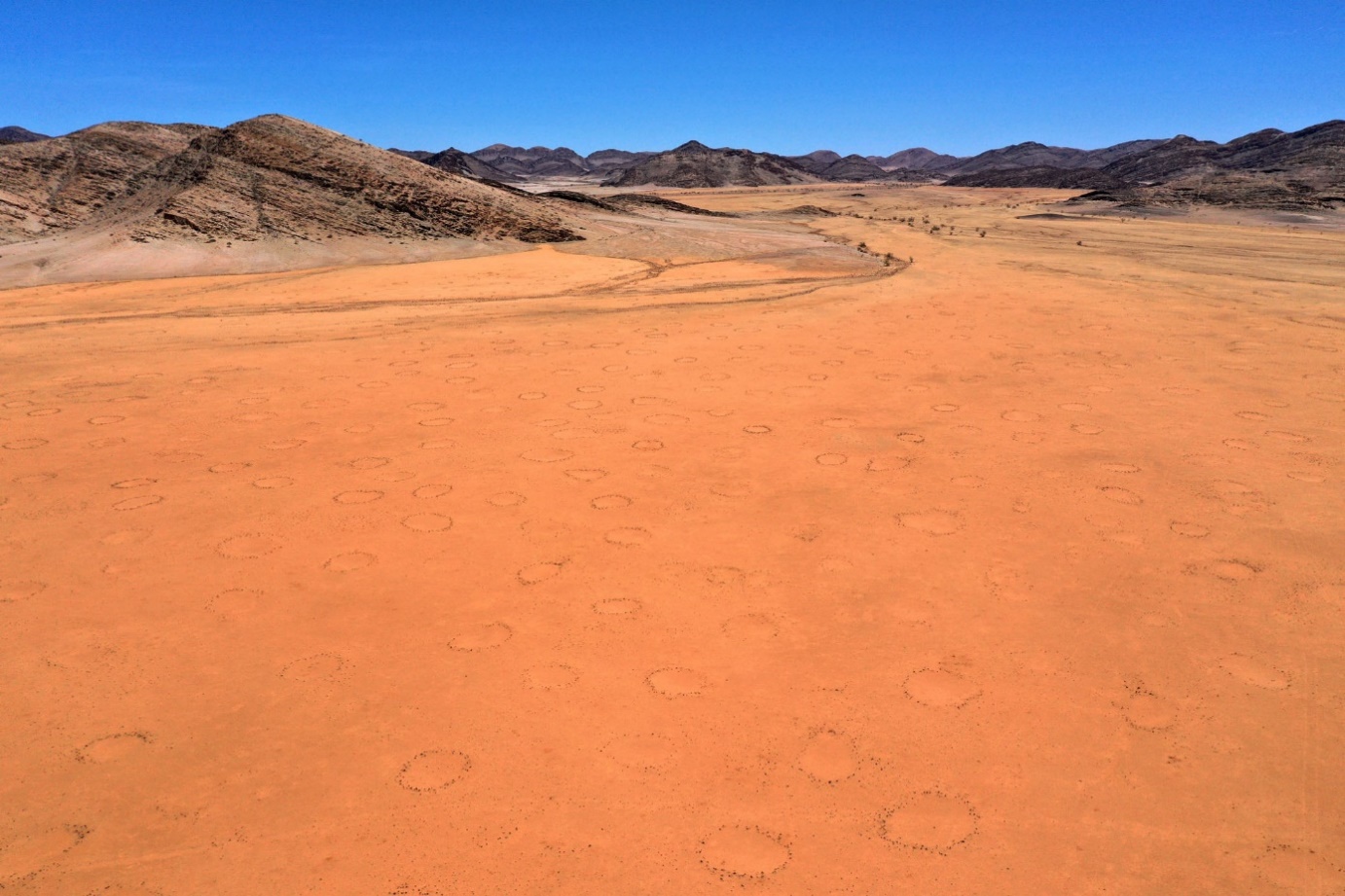


**Fig. S5** This drone image shows the “C1-plot”, as used for spatial analysis of FCs, by Meyer et al. [14]. This plot, however, is not representative for the spatially periodic ordering of FCs that can be seen in vast parts of the Giribes Plains further north under more homogeneous habitat conditions. The C1-plot shown here is directly affected by the drainage line in the background because this drainage line will channel away the run-off water that flows down from the mountain towards the C1-plot. The same applies to the drainage channel on the right side. All this habitat heterogeneity leads to a non-homogeneous distribution of rain water in this C1-plot, which lowers the spatial ordering of the FCs inside the plot. Water is the main driver in such arid systems and any disruption in topographic homogeneity leads to less ordering.


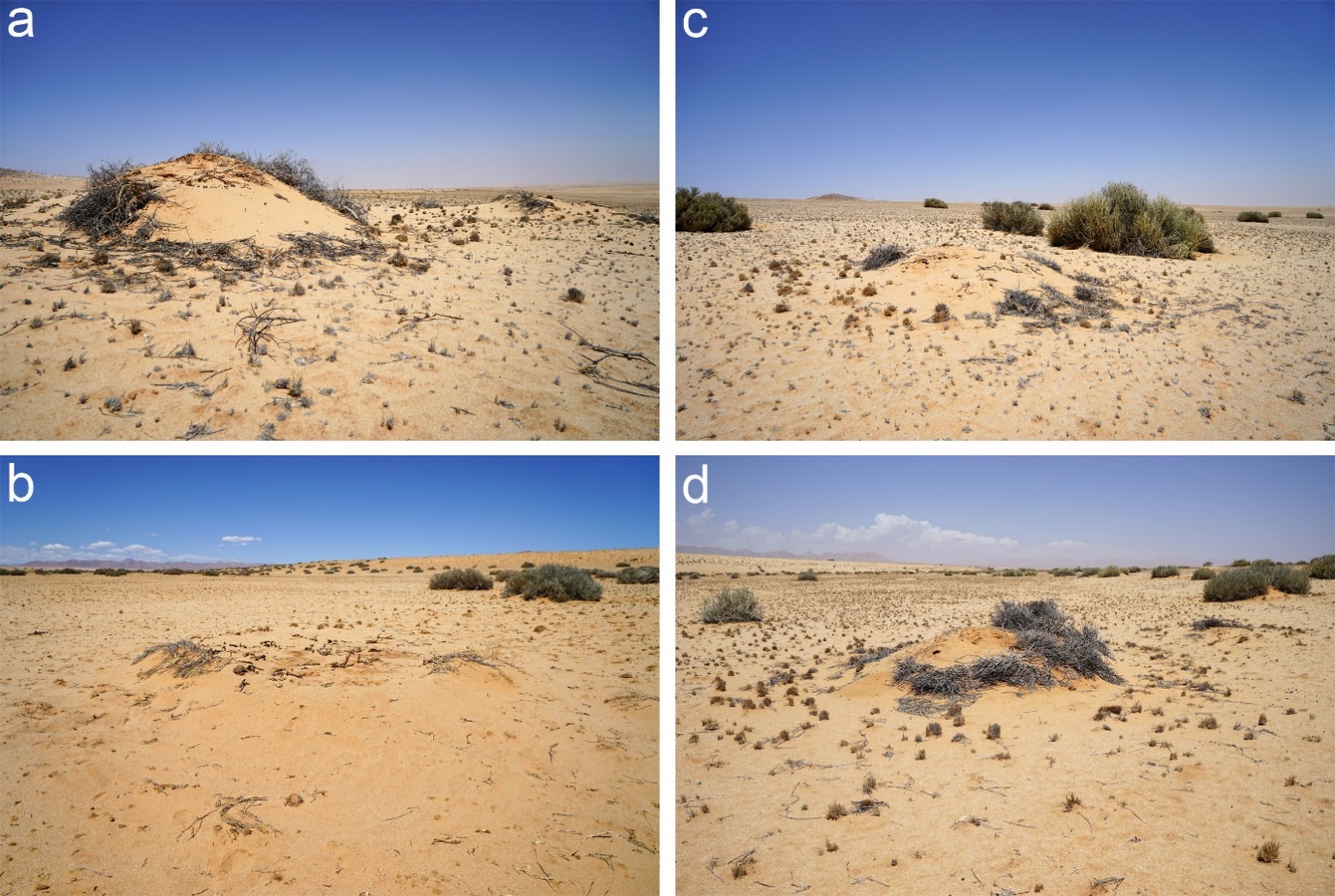


**Fig. S6** Several examples of typical sand piles, accumulating around decaying *Euphorbia gummifera* at the two sites Gar-1 and Gar-2 near Garub (a-d). These circular sand piles with reduced vegetation cover may look similar to fairy circles in aerial or satellite imagery. However, these piles of sand cannot be called fairy circles.


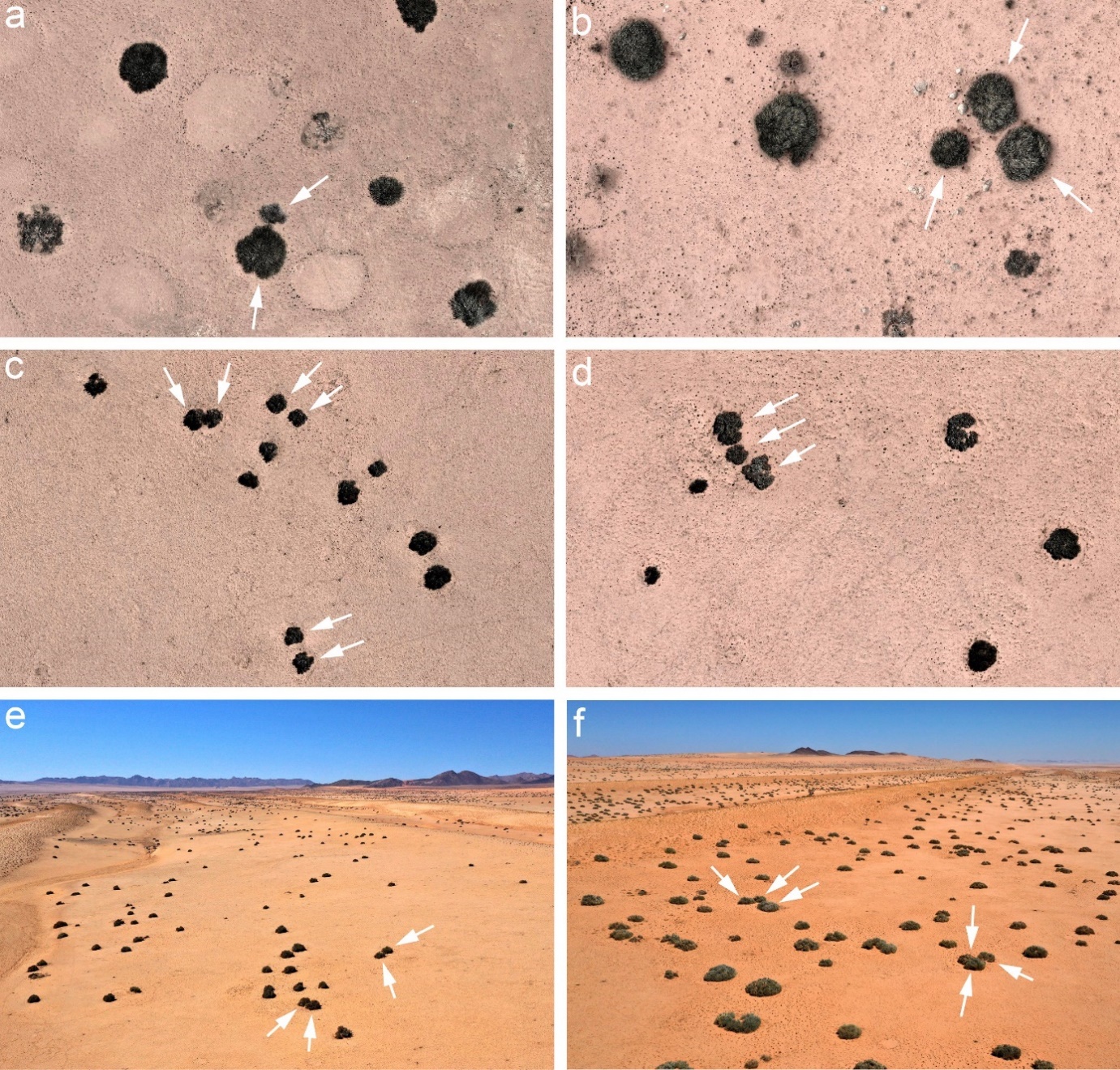


**Fig. S7** Evidence for clustered patterns of *E. damarana* and *E. gummifera*. *Euphorbia damarana* in the Theron-plot Gir-1 of the southern Giribes (a) and in the plot Bra-2 near Brandberg (b) showed statistically significant clustering because regeneration of the shrubs often happens in a clumped distribution where individual shrubs are tightly packed together (arrows). Also, *Euphorbia gummifera* in the plot Gar-1 near Garub showed strongly clustered patterns (c) while in the plot Gar-2 the Euphorbias showed a mixed pattern of clustered and random distributions (d). The clustering results from individual shrubs growing next to each other (arrows) as the high-resolution ortho-images from drone mapping demonstrate. Such a pattern is in contrast to the distribution of FCs because in many FC areas such as the Giribes, the Brandberg, Marienfluss Valley or the NamibRand Nature Reserve, FCs have typically no neighbors in their immediate neighborhood. Also, oblique drone images with overviews over the plots Gar-1 (e) and Gar-2 (f) near Garub clearly show how the individual shrubs with their humped growth form are tightly clustering but they do not have a regularly spaced pattern.


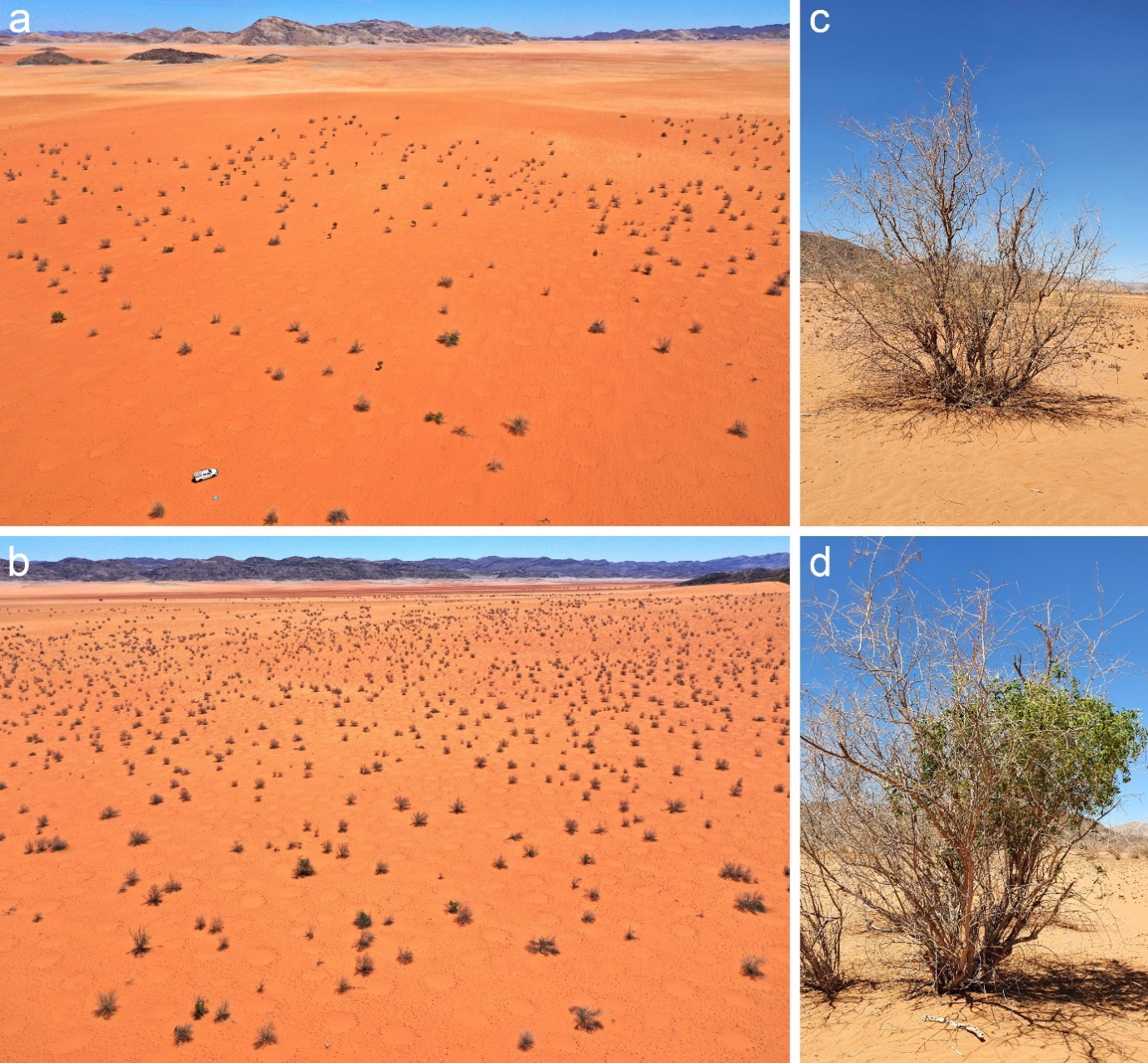


**Fig. S8** In the north-eastern part of the Giribes Plains, the species *Parkinsonia africana* co-occurs in various densities with fairy circles over an area of several square kilometers. Two examples have been photographed in March 2020 with a drone (a,b). *Parkinsonia africana* is a shrub species or small tree, which may grow on its own (c) or it may be occupied by *Salvadora persica* which scrambles into it (d).
